# An integrin beta-1-anchored bicaudal C1-polycystin-1 module essential for tubular morphogenesis in polycystic kidney disease

**DOI:** 10.1016/j.isci.2026.117209

**Published:** 2026-08-17

**Authors:** Andrew J. Streets, Manoj K. Valluru, Devon Smith, Lisa Chang, Iddo Z. Ben-Dov, Stuart A. Wilson, Christopher P. Toseland, Albert C.M. Ong

**Affiliations:** 1 Kidney Genetics Group, Division of Clinical Medicine, School of Medicine and Population Health, University of Sheffield, Sheffield, UK; 2 Laboratory of RNA Molecular Biology, The Rockefeller University, New York, NY, USA; 3 School of Biosciences, University of Sheffield, Sheffield, UK; 4 Sheffield Institute for Nucleic Acids, University of Sheffield, Sheffield, UK; 5 Cancer Biophysics Group, Division of Clinical Medicine, School of Medicine and Population Health, University of Sheffield, Sheffield, UK

**Keywords:** PKD, kidney tubule, polycystin-1, bicaudal C1, RNA binding proteins, F-actin, focal adhesions, integrin b1, FBLMI1, genetics

## Abstract

Genetic variants in the RNA-binding protein bicaudal C1 (BICC1) have been linked to very-early-onset polycystic kidney disease (PKD). This study identifies a crucial integrin-beta1 (ITGB1)-BICC1-polycystin-1 (PC1) complex that regulates F-actin organization and cell adhesion in human kidney cells through the expression of filamin-binding-LIM-protein-1 (FBLIM1). Both *Bicc1* and *Pkd1* knockout cells exhibit abnormal F-actin structure, increased cell stiffness, decreased adhesion and impaired migration while inhibiting F-actin branching directly triggers cyst formation. Locally, BICC1 interacts with F-actin, ITGB1, and FBLIM1 at focal adhesions. ITGB1 knockdown reduces surface BICC1 expression. In turn, BICC1 stabilizes FBLIM1 by influencing transcript abundance, promoting its local translation, and protecting it from degradation in the presence of PC1. In summary, these findings reveal a novel model of cystogenesis linking BICC1’s dual RNA and protein regulatory functions to the maintenance of cytoskeletal and tubular integrity.

## Highlights

• BICC1 is expressed in multiple subcellular compartments, indicating diverse cellular functions
• A beta1-integrin-BICC1-polycystin-1 module regulates F-actin organization through FBLIM1
• BICC1 and PC1 stabilize the expression of each other and of FBLIM1
• A cilia-independent model of cyst formation around focal adhesion integrity is identified

## Graphical abstract

**Figure:**
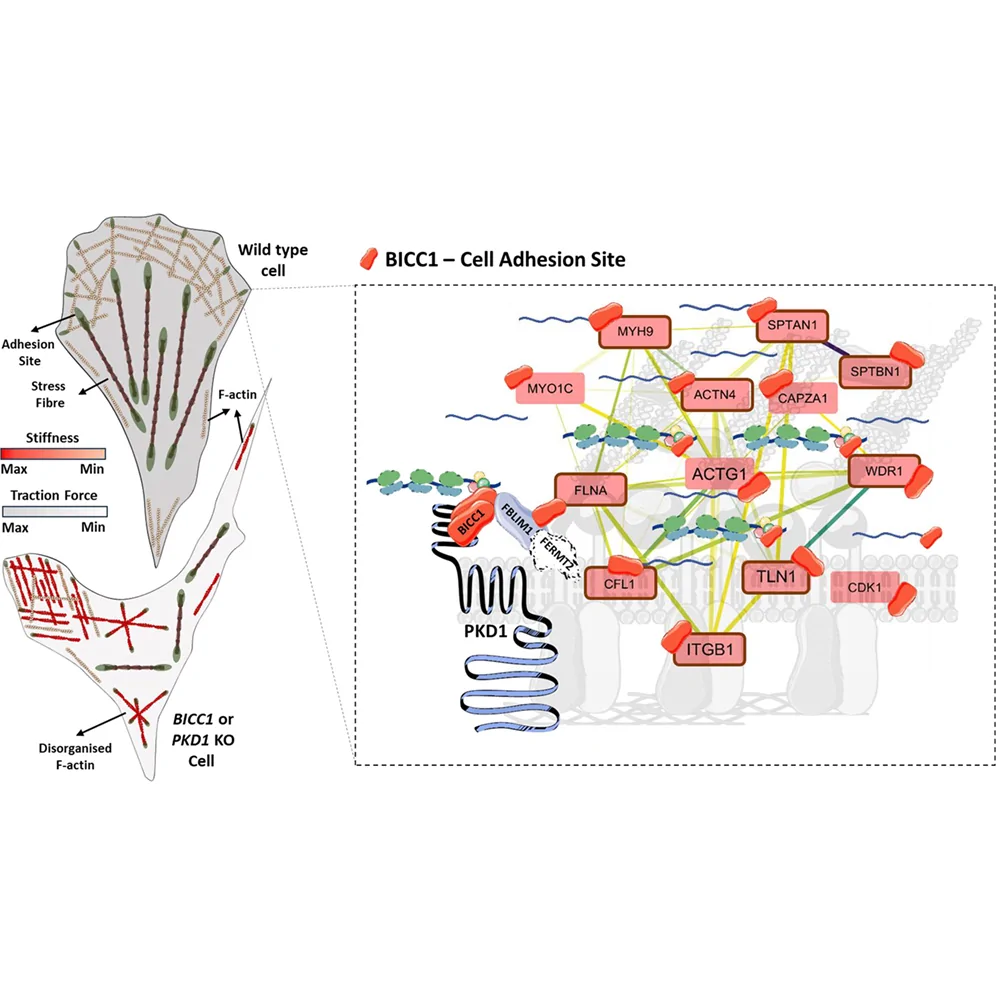

## Teaser

Nephrology; Mechanobiology; Cell biology

## Introduction

Autosomal dominant polycystic kidney disease (ADPKD) is the most common monogenic kidney disease and a leading cause of kidney failure.^1^ Variants in *PKD1* (70%–80%) or *PKD2* (10%–20%), encoding polycystin 1 (PC1) and polycystin 2 (PC2), account for over 90% of cases, with several minor PKD genes accounting for less than 5%. Although the polycystin proteins were first identified over 25 years ago, a universal consensus on their physiological functions and how *PKD1* or *PKD2* mutations result in cyst formation is still lacking. Nevertheless, there is increasing evidence for a common cystogenic pathway for all forms of ADPKD centered around PC1 expression or function.^2^

Bicaudal-C family RNA-binding protein 1 (BICC1) was first identified in *Drosophila melanogaster BicC* mutants with double abdomens due to defects in antero-posterior body-axis specification.^3^ It is a highly evolutionarily conserved RNA-binding protein with 5 K-homology (KH) or K-homology like (KHL) RNA-binding domains and a C-terminal sterile alpha motif (SAM) domain. *Bicc1* variants were subsequently shown to result in a neonatal recessive PKD phenotype in two allelic mouse models (*bpk* and *jcpk*).^4^ Heterozygous *BICC1* variants have also been reported in two infants with cystic dysplasia, although both variants were also present in an unaffected parent.^5^

In a recent study, we reported BICC1 as a novel binding partner of PC1 and PC2 and found evidence of a genetic interaction in *Xenopus*, mice, and humans.^6^ Specifically, homozygous *BICC1* or digenic *BICC1* and *PKD1 (*or *PKD2)* variants were associated with a very-early-onset (VEO) PKD phenotype. The major aim of this study was to investigate the molecular mechanisms driving cystogenesis that result from *BICC1* variants and how they relate functionally to the major ADPKD protein, PC1.

## Results

### Normal F-actin organization is dependent on BICC1 or PC1 expression and correlates with normal tubulogenesis

Single-cell RNA sequencing studies indicate that *BICC1* is expressed by human kidney tubular epithelial cells with the highest expression seen in more proximal nephron segments i.e., S2, S3, LOH DL (loop of Henle descending limb), and parietal epithelial cells (Figure S1). Since BICC1 is known to oligomerize via its SAM domain,^7^ we reasoned that ectopic expression of Bicc1 in wild-type cells might lead to dominant-negative effects on cellular function and that inducible re-expression in a *BICC1* null background would avoid this possibility. We, therefore, utilized an established conditionally immortalized human proximal tubular cell line (UCL93) as the main cellular model to study the cellular phenotype of *BICC1* loss-of-function and its interaction with *PKD1* in an isogenic background.^8^ This line expresses a gene signature characteristic of the proximal tubular S3 segment (Figure S2).^9^

**Figure 1:**
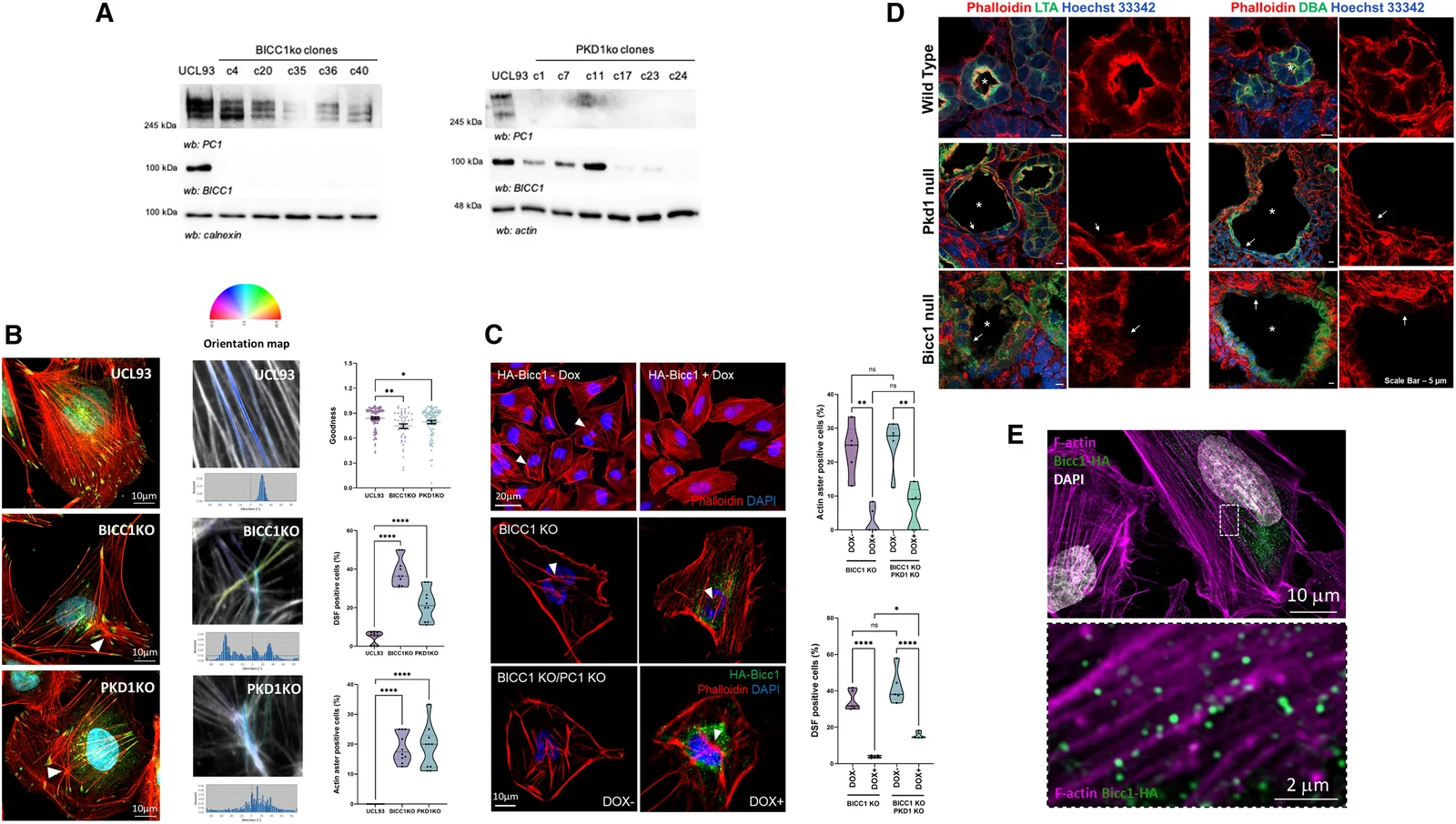
BICC1 and PC1 show reciprocal co-expression and regulate F-actin stress fiber organization (A) Western blot analysis for PC1 or BICC1 in selected BICC1 and PKD1 KO clones (*n* = 5). PC1 expression was reduced in *BICC1* KO cells and BICC1 expression reduced in *PKD1* KO cells. Expression was normalized to calnexin (quantitation in Figure S1B). (B) F-actin stress fiber organization in UCL93, *PKD1* KO, and *BICC1* KO Cells. The left image displays phalloidin (red) and paxillin (PXN, green) stained cells, illustrating normal actin stress fibers in UCL93 cells and disordered stress fibers (DSF) and actin asters (arrowheads) in KO cells. The right image features orientation map images and directionality histograms, indicating the directionality of actin stress fibers with a broader angular dispersion in KO cells compared with UCL93 cells. Dot plots show that the goodness of Gaussian fit for directionality is significantly higher in UCL93 cells relative to KO cells. Each data point is region of interest (ROI) of a single cell (UCL93 *n* = 94, *BICC1*KO *n* = 47, and *PKD1*KO *n* = 89 cells). Violin plots reveal a high proportion of DSF and actin asters in KO cells compared with UCL93 cells. Each data point is representative of a captured ROI with approximately *n* = 14–20 cells per ROI (UCL93 *n* = 134, *BICC1* KO *n* = 339, and *PKD1* KO *n* = 143 cells). Scale bars, 10 μm. (C) Representative images of phalloidin stained *BICC1* KO and *BICC1*/*PKD1* KO cells before or after doxycycline induction (+/−DOX). Scale bars, 20 μm. Inducible expression of HA-Bicc1 completely rescued the actin phenotype in *BICC1* KO cells but not in double KO cells, as shown in the violin plots. Scale bars, 10 μm. Each data point is representative of a captured ROI with approximately *n* = 10 cells per ROI and a total of *n* = 43–52 cells per condition. Data are presented as mean values ± SEM, one-way ANOVA was used for statistical analysis with a *p* value of <0.05 indicating statistical significance (∗*p* ≤ 0.05, ∗∗*p* ≤ 0.01, ∗∗∗*p* ≤ 0.001, and ∗∗∗∗*p* ≤ 0.0001). (D) In normal tubules from wild-type mice, cortical F-actin can be visualized apically and basally. This organization is severely disrupted in both proximal (LTA) and distal (DBA) cystic segments of Pkd1 and Bicc1 null kidneys. Samples were triple-stained for F-actin (phalloidin, red), LTA (green), DBA (green), and nuclei (Hoechst 33342). Individual cysts are indicated (∗) and focal areas of loss of apical F-actin are indicated by arrows. Scale bars, 5 μm. (E) Co-localization of HA-Bicc1 along F-actin stress fibers (phalloidin-positive) sites in *BICC1* KO cells after induction of HA-Bicc1 expression. Scale bars, 10 μm. The bottom image is an expanded view of the selected ROI (boxed). Scale bars, 2 μm.

*BICC1* null and double *BICC1/PKD1* null cells were first generated by CRISPR-Cas9 editing of UCL93 cells, as previously described for *PKD1*.^8^ A decrease in BICC1 expression was observed in *PKD1* null cells as previously reported.^10^ Conversely, PC1 expression was also lower in *BICC1* null cells which could imply mutual stabilization between both proteins (Figures 1A and S3A). We also observed striking disorganization of F-actin stress fibers in *BICC1* and *PKD1* knockout (KO) lines characterized by loss of the normal parallel fiber orientation and neo-formation of actin “aster” structures (Figure 1B). This phenotype was directly related to loss of BICC1, as it was completely rescued by doxycycline-inducible lentiviral expression of epitope-tagged exogenous mouse Bicc1 (Figures 1C, S3B, and S3C). However, a partial rescue was observed in *BICC1/PKD1* null cells, implying that PC1 was essential for the actin phenotype (Figure 1C). The actin phenotype was observed in both sub-confluent and confluent monolayers (Figure S4).

To determine the *in vivo* relevance of these findings, we studied F-actin distribution in wild-type and *Pkd1* and *Bicc1* mutant mouse kidneys.^8^^,^^11^ In wild-type mice, F-actin was localized at the apical and basal surfaces of kidney tubules (Figure 1D).^12^ In *Pkd1* and *Bicc1* mutant kidneys, cysts arose from both proximal (lotus tetragonobolus lectin or LTA positive) and distal (dolichos biflorus agglutinin lectin or DBA positive) segments, though many larger cysts did not stain for either marker (not shown). Prominent disruption of apical F-actin staining was observed in both LTA and DBA positive small cysts, starting in a discontinuous manner in smaller cysts with complete loss in larger cysts (Figure 1D).

To investigate whether disruption of F-actin organization alone was sufficient to interfere with normal tubulogenesis, we utilized UCL93 cells, which normally form branching tubular structures in 3D culture.^13^ Inhibition of F-actin branching using the ARP2/3 inhibitor CK-666^14^ induced the striking formation of cystic structures (Figure S3D). This suggested that defects in the organization of the actin cytoskeleton can lead directly to cyst formation *in vitro*.

### The subcellular localization of BICC1 in human kidney epithelial cells

The localization of endogenous BICC1 in human kidney epithelial cells has not been previously reported. Studies of heterologous murine Bicc1-GFP in HEK-293 and HeLa cells had revealed striking co-localization to cytoplasmic granules, partially overlapping with markers of p-bodies and stress granules.^15^^,^^16^ Similarly, we detected endogenous BICC1 in cytoplasmic granules in UCL93 cells (Figure S5A).

In our study, endogenous BICC1 also localized to other intracellular compartments, including paxillin (PXN) and beta1-integrin (ITGB1) positive focal adhesion (FA) sites, F-actin stress fibers (Figures 1E, S5B, and S5C), and centrosomes. The specificity of the BICC1 antibody for immunofluorescence (IF) detection is shown by its absence in *BICC1* null cells and its expression after induction (Figure S3B).

Using a phalloidin pull-down assay, we confirmed that endogenous BICC1 could interact with F-actin and this interaction is mediated by its N-terminal domain (aa 1‑132) (Figures S6A–S6C) indicating its likely specificity. There was no localization to primary cilia but endogenous BICC1 and stably expressed HA-Bicc1 localized prominently to pericentrin-labeled centrosomes (Figures S7A and S7B). There was no evidence of a structural ciliary defect, expressed as changes in cilia length or percentage of ciliated cells (Figures S7C and S7E). Nonetheless, a subtle ciliary functional defect could not be excluded since the mean orientation angle of the cilia shaft was altered in *BICC1* KO cells compared to controls (Figure S7D) as has been reported for nodal cilia in *Bicc1* null mice, correlating with changes in planar cell polarity.^16^

### *BICC1* and *PKD1* null cells share common defects in cell adhesion and cell migration indicative of altered FA function

**Figure 2:**
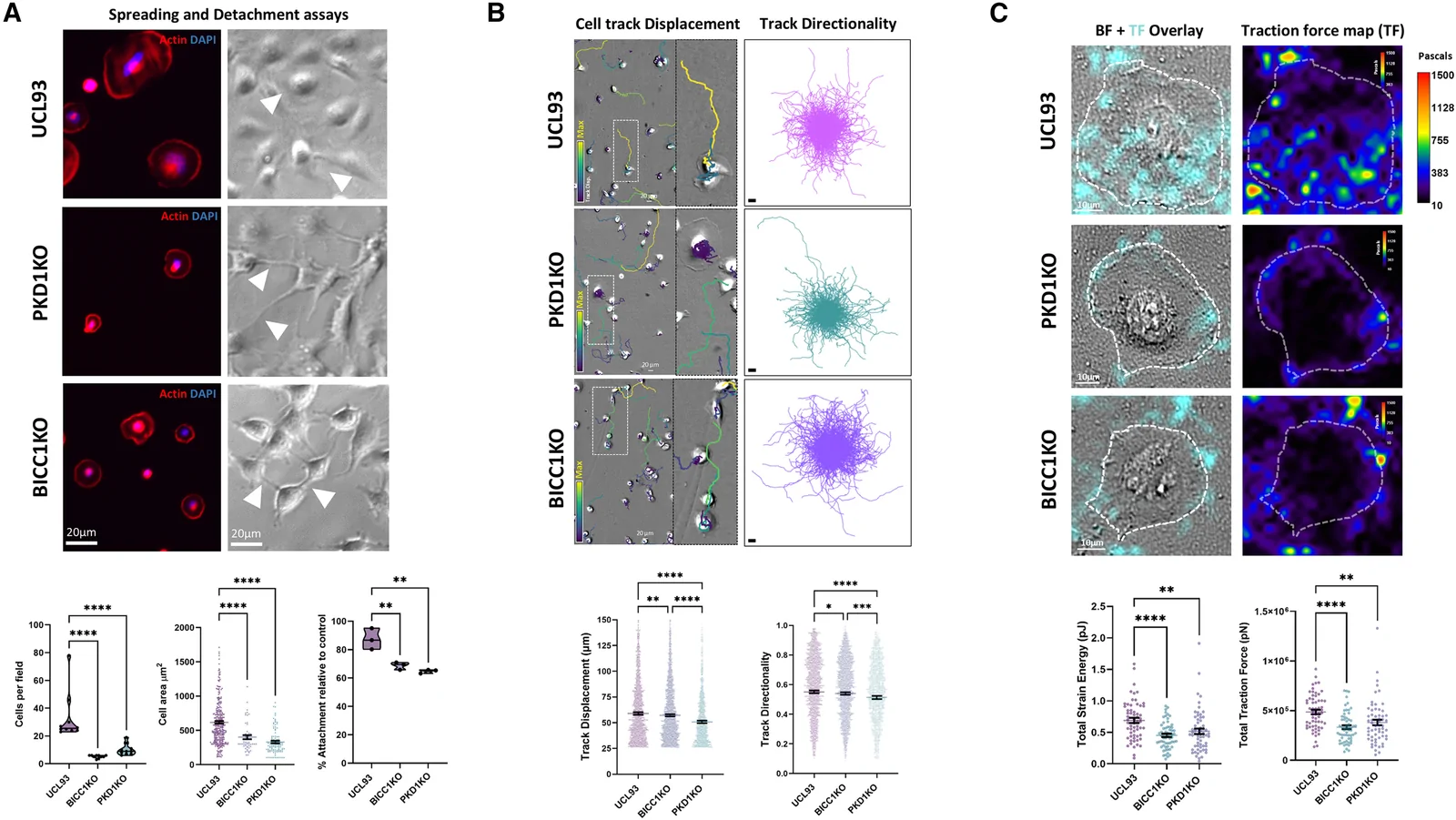
Defects in cell adhesion, cell migration, and FA function are common to *BICC1* and *PKD1* null cells (A) *BICC1* and *PKD1* KO cells show significantly reduced cell number (*n* = 3 independent experiments, *N* = 10 individual fields) and overall attached cell area (*n* = 3 independent experiments, *N* > 100 cells individual cells) after 30 min attachment. Following 30 min incubation with 1 mM EDTA, *BICC1* and *PKD1* KO cells showed significantly fewer adherent cells after washout with PBS (*n* = 3 independent experiments, *N* = 3 replicates). Scale bars, 20 μm. (B) Representative single-cell track displacement and track directionality images of UCL93, *PKD1* KO, and *BICC1* KO cells demonstrating altered migration in the KO cells. Dot plots showing a significant decrease in track displacement (μm) and track directionality (net-displacement/total-distance) of KOs relative to UL93 cells (UCL93 *n* = 2,043, *BICC1* KO *n* = 2,538, and *PKD1* KO *n* = 1,407 cell-tracks from *n* = 3 biological replicates). Scale bars, 20 μm. (C) Representative traction force maps of UCL93, *PKD1* KO, and *BICC1* KO cells, with a color key indicating the magnitude of traction force, in pascals. Dot plots showing a significant decrease in total strain energy (pJ) and total traction force (pN) of KOs relative to UL93 cells (*n* = 60 cells/line from 3 biological replicates). Scale bars, 10 μm. One-way ANOVA was performed to assess statistical significance (∗*p* < 0.05, ∗∗*p* < 0.01, ∗∗∗*p* < 0.001, ∗∗∗∗*p* < 0.0001).

Compared with wild-type cells (UCL93), both *PKD1* and *BICC1* null cell lines showed profound defects in cell attachment, spreading, and detachment (Figure 2A). *BICC1* and *PKD1* null cells also displayed significantly impaired motility in single-cell migration assays, as measured by a reduction in track displacement (mean differences of 3.31 and 10.64 μm, respectively) and track directionality (net displacement/total distance) (12 h, *p* < 0.05) (Figure 2B). To understand this phenotype better, we characterized the spatial biomechanical signature by using atomic force microscopy (AFM) with PeakForce quantitative nanomechanics (QNM) and PeakForce Tapping. *PKD1* null cells displayed a significant increase in cell stiffness compared with UCL93 cells, while *BICC1* null cells showed a similar trend (Figure S8). Moreover, in agreement with the actin structures seen in Figure 1, disorganized dense and stiff actin structures were clearly visible in the nanomechanical maps. Finally, traction force microscopy demonstrated that the null cells displayed reduced levels of strain energy and traction forces when compared with wild type (*p* < 0.05) (Figure 2C).

Together, these observations indicate that cell adhesion, the capacity to exert contractile forces on the surrounding environment, and migration were all decreased in null cells. This may be due to impaired FA formation or stability and actin organization, which in turn increases cell stiffness. However, in view of the lower traction forces, the filaments are probably non-contractile.

### Mass spectrometry of HA-BICC1 identified ITGB1 and components of FA and actin regulators

**Figure 3:**
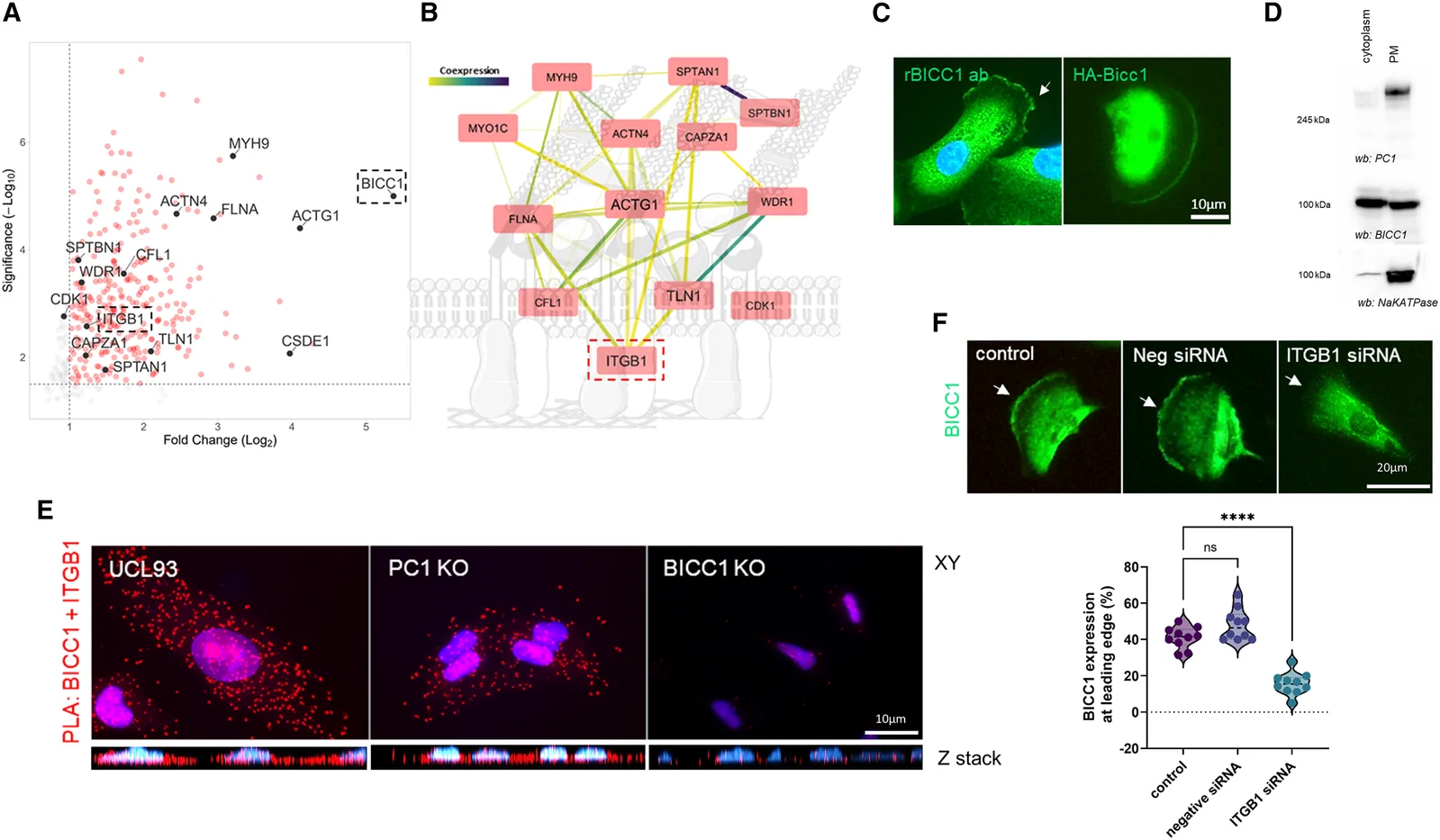
A plasma membrane fraction of BICC1 interacts with ITGB1 and other interacting proteins associated with FAs or actin regulation identified by LC-MS/MS pull-down of HA-BICC1 (A) Mass spectrometry identification of proteins co-purified with HA-BICC1 (coIP controls *n* = 3 and HA-BICC1 *n* = 3). A scatterplot displays significantly enriched proteins co-purified with the bait HA-BICC1 (*n* = 356), highlighting several cell adhesion proteins (*n* = 13). (B) STRING network of HA-BICC1 interacting cell adhesion proteins depicts protein-protein associations, including the FA protein ITGB1. Edges are color-coded for coexpression, and their thickness indicate the confidence score. (C) BICC1 was localized to the leading edge in UCL93 cells following doxycycline induction of HA-Bicc1. Left image shows labeling with a polyclonal BICC1 antibody and the right image with an anti-HA epitope antibody. Scale bars, 10 μm. (D) Membrane fractionation assay demonstrating that endogenous BICC1 was expressed both in the cytoplasm and at the plasma membrane in UCL93 cells. PC1 was only detected in the plasma membrane fraction where Na^+^ K^+^ ATPase is enriched. (E) Proximity ligation assay (PLA) demonstrates that endogenous BICC1 and ITGB1 interact at the basolateral surface of UCL93 cells. Notably, a z stack maximum projection image shows a strong PLA signal on the basal side of cells (YZ). There was no difference in the PLA signal in the absence of PC1. As expected*, BICC1* KO cells showed a low background signal. Scale bars, 10 μm. (F) Surface BICC1 localization was significantly reduced in cells transfected with ITGB1 siRNA compared with scrambled siRNA or un-transfected cells. (*n* = 3 independent experiments, *N* = 10 individual fields per treatment condition). Scale bars, 20 μm. The violin plot indicates a significant reduction in BICC1 expression at the leading edge in *ITGB1* knockdown cells compared with control and scrambled (neg) siRNA treated cells. One-way ANOVA was performed to assess statistical significance (∗∗∗∗*p* < 0.0001 and ns, not significant).

To investigate the molecular basis of these changes, we conducted a pull-down of epitope-tagged HA-BICC1 from stably transformed HEK-293T-Rex cells and analyzed its interactome by mass spectrometry (MS) (Figure 3A). A total of 356 proteins were identified with high stringency (*p* < 0.05), mapping to 329 gene sets, which included previously reported candidates i.e., ANKS3, CAD, FASN, and several CCR4-NOT deadenylase subunits.^17^ In addition, several cell adhesion cluster proteins were co-purified, notably β1-integrin (ITGB1), talin1 (TLN1), monomeric or polymeric actin (ACTG1), and a range of actin regulatory proteins involved in branching (FLNA), bundling (ACTN4), capping (CAPZA1), disassembly (CFL1 and WDR1), and contractility (MYH9, MYO1C, SPTAN1, and SPTBN1) (Figure 3B and Data S1).

In migrating UCL93 cells, a surface fraction of endogenous BICC1 or HA-Bicc1 was clearly visible at the leading edge by IF, where it co-localized with ITGB1 (Figure 3C). Due to the lack of a suitable PC1 antibody for IF, we employed membrane fractionation and detected a significant fraction of endogenous BICC1 co-sedimenting with PC1 in the plasma membrane (PM) (Figure 3D), confirming our recent observations that they are interacting partners.^18^ Unlike PC1 however, BICC1 was almost equally distributed between cytoplasmic and PM fractions. We also confirmed the interaction of endogenous BICC1 with ITGB1 by proximity ligation assays (PLAs) at the basolateral membrane (Figure 3E) and found that small interfering RNA (siRNA) knockdown of ITGB1 significantly reduced BICC1 surface expression (Figure 3F). Surface BICC1 could, however, still be visualized in *PKD1* null cells indicating that PC1 was not essential for BICC1 PM localization (Figure 3E).

### RNA sequencing identifies a common gene signature implicating changes in cell junction organization, adhesion, and motility in *BICC1* and *PKD1* null cells

**Figure 4:**
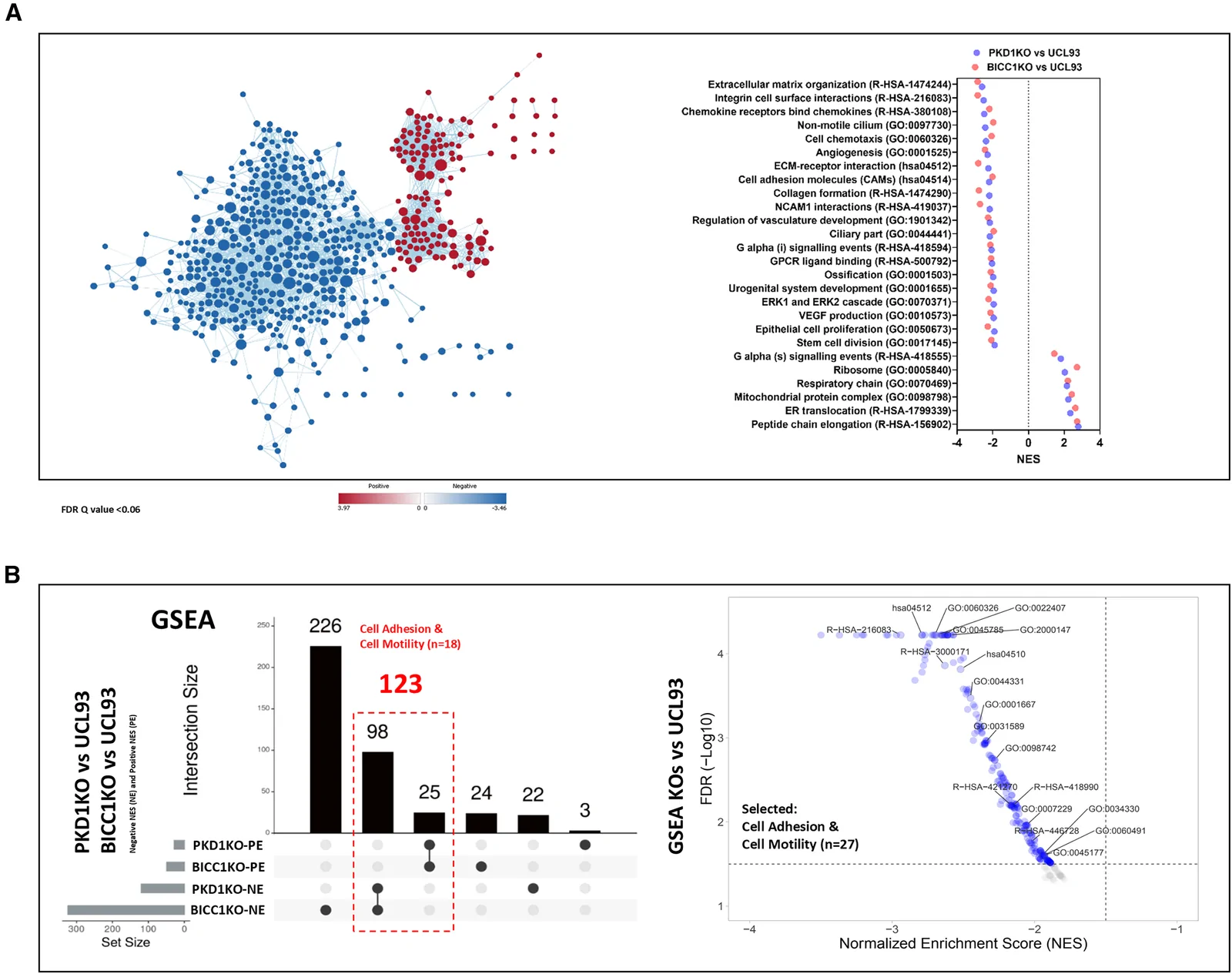
Gene set enrichment analysis and common enriched gene sets in *BICC1* and *PKD1* null cells and curation of an actin associated proteins gene set (A) The GSEA output enrichment map network shows significantly enriched gene sets in both KO cells compared with UCL93 cells, with 263 negative NES gene sets and 81 positive NES gene sets. The plot highlights 26 significantly enriched gene sets in common between *BICC1* KO vs. UCL93 and *PKD1* KO vs. UCL93. (B) The UpSet plot illustrates the significant common enriched gene sets (PE, positive NES and NE, negative NES) between *BICC1* KO vs. UCL93 and *PKD1* KO vs. UCL93, highlighting 123 common gene sets, with 18 associated with cell adhesion and cell motility. The scatterplot shows 27 cell adhesion and cell motility-associated gene sets negatively enriched in the KO cells compared with UCL93 cells.

**Figure 5:**
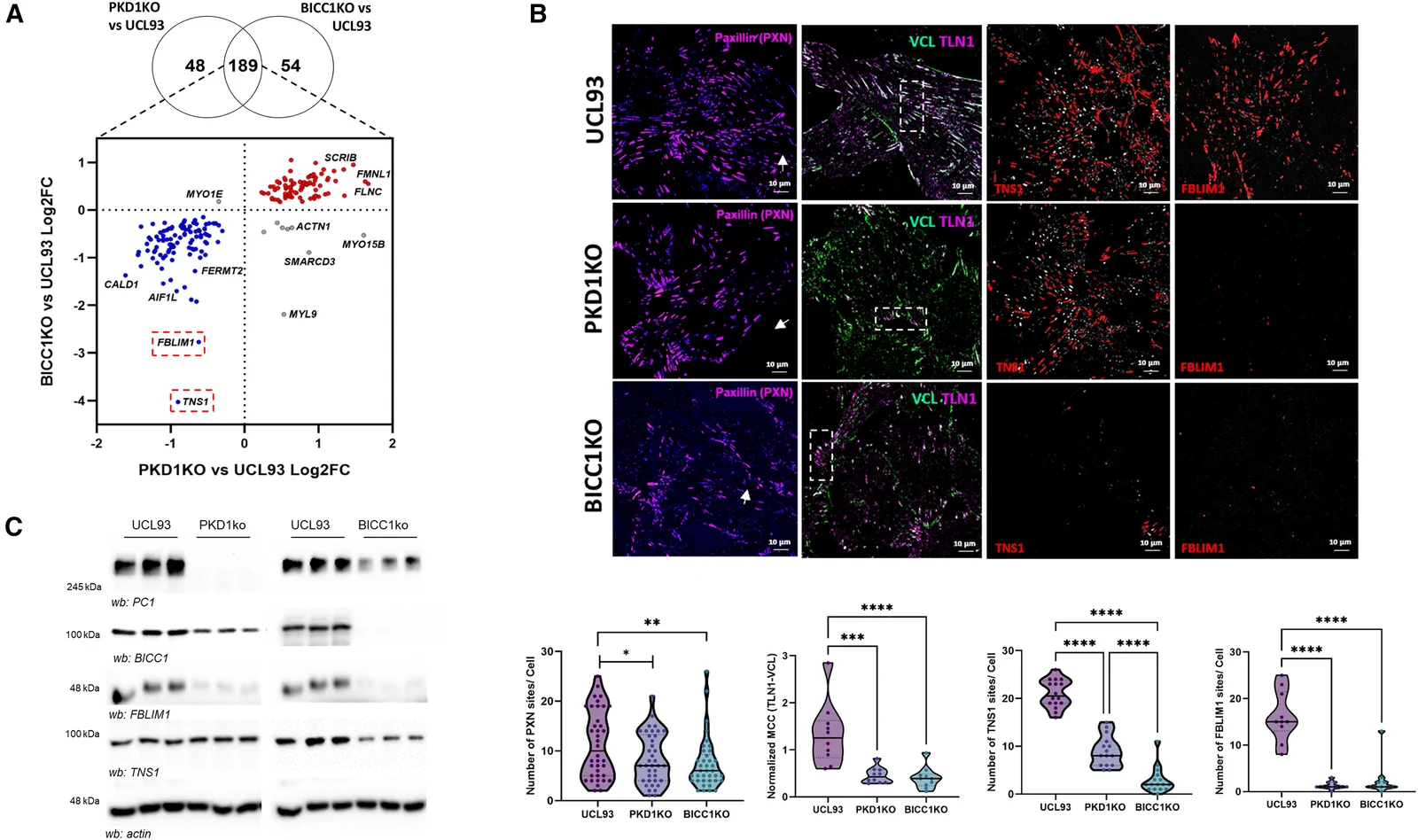
RNA sequencing reveals a common disease gene signature in *BICC1* and *PKD1* null cells which correlates with changes in FA number and stability (A) Venn diagram and scatterplot of differentially expressed genes for actin-associated proteins (AAPs). Curation of these AAPs is shown in Figure S6. The Venn diagram shows 189 AAPs differentially expressed in both *BICC1* KO and *PKD1* KO (*p* < 0.05). The scatterplot displays the distribution of these 189 AAPs with Log2 fold change, highlighting upregulated (red) and downregulated (blue) candidates. The boxes indicate the top hits, *TNS1* and *FBLIM1*. (B) Immunolabelling of FA sites by PXN (left), TLN1 and VCL (middle), and TNS1 and FBLIM1 (right). Reduced FA sites were found in KO cells by PXN staining (red). Each data point is representative of a captured ROI with approximately *n* = 9–12 cells per ROI, *n* = 38 ROIs/line (UCL93 *n* = 418, *PKD1* KO *n* = 405, and *BICC1* KO *n* = 347 cells from 3 biological replicates). Reduced co-localization of VCL (green) and TLN1 (red) was observed in KO cells (arrowheads). Each data point is representative of a captured ROI with approximately *n* = 6–8 cells per ROI, (UCL93 *n* = 74, *PKD1* KO *n* = 67, and *BICC1* KO *n* = 60 cells). A significant reduction in both TNS1 and FBLIM1 FA sites was detectable in KO cells. Each data point is representative of a captured ROI: TNS1 with approximately *n* = 15 cells per ROI (UCL93 = 324, *PKD1*KO = 230, and *BICC1* KO = 299 cells), FBLIM1 with approximately *n* = 9–15 cells per ROI (UCL93 = 101, *PKD1*KO = 85, and *BICC1* KO = 147 cells) from three independent experiments. Violin plots indicate overall data points obtained from each line. Scale bars, 10 μm. (C) Western blots showing decreased FBLIM1 expression in both *BICC1* and *PKD1* KO clones (*n* = 3). TNS1 was reduced in *BICC1* KO clones only. Actin was used as a loading control. Data are presented as representative blot of 3 separate repeat experiments.

Since BICC1 is an RNA-binding protein that has been shown to regulate mRNA stability and translation,^15^^,^^16^^,^^19^ we performed RNA sequencing in *BICC1* and *PKD1* null cells, searching for common gene clusters or networks that could be regulated by BICC1 and PC1. Gene set enrichment analysis (GSEA) identified 123 common dysregulated gene sets in both cell lines, notably ECM-receptor interaction, cell chemotaxis, and integrin cell surface interactions (Figures 4A and 4B). To understand this better, we curated a list of actin-associated proteins (AAPs) from several published datasets (*n* = 431, Figure S9). Interestingly, 189 AAP genes were dysregulated in both *BICC1* and *PKD1* null cells (Figure 5A). Moreover, the top 21 of these dysregulated AAPs were associated with cell adhesion complexes. In particular, tensin1 (*TNS1*), migfilin (*FBLIM1*), and kindlin-2 (*FERMT2*), known to be critical for the formation and stability of FAs, were significantly decreased in both cell lines^20^^,^^21^ (Figure 5A). Indeed, a *Tns1* KO mouse was previously reported to develop glomerular and proximal tubular cysts.^22^ Western blot analysis confirmed that the expression of FBLIM1 was greatly reduced in multiple *BICC1* and *PKD1* null clones (Figures 5C, S10A, and S10B). Despite the reduction in *TNS1*, TNS1 protein expression was reduced in *BICC1* null but not *PKD1* null cells (Figures S10A and S10B).

We next investigated whether these changes correlated with alterations in FA number or phenotype. The recruitment of vinculin to talin at FA sites is force-dependent, locking talin into an actin-binding configuration which in turn stabilizes FA.^23^^,^^24^ Notably, both FA number (paxillin positive) and stability (vinculin/talin double-positive) were significantly reduced in *BICC1* and *PKD1* null cells (Figure 5B). We also detected significantly fewer TNS1 and FBLIM1 positive FA sites in *BICC1* and *PKD1* null cells (Figure 5B).

### BICC1 binds to endogenous *FBLIM1* mRNA by RNA-IP and regulates its polarized translation

Finally, to explore whether BICC1 could be regulating the translation or stability of these mRNAs, we performed RNA-immunoprecipitation (RNA-IP). Indeed, novel BICC1 mRNA targets (*PKD1*, *TNS1*, and *FBLIM1*) as well as known mRNA targets (*PKD2* and *BICC1*) were found to bind endogenous BICC1 (Figure S11). Of interest, previous studies in *Drosophila* and *Xenopus* oocytes revealed several BicC targets involved in cytoskeletal organization (gamma actin, actin organization, dynamics, and cell adhesion), though these have not been investigated in mammalian cells.^25^^,^^26^

### Re-expression of FBLIM1 in *BICC1*, *PKD1*, and *BICC1/PKD1* null cells completely rescued the F-actin phenotype

**Figure 6:**
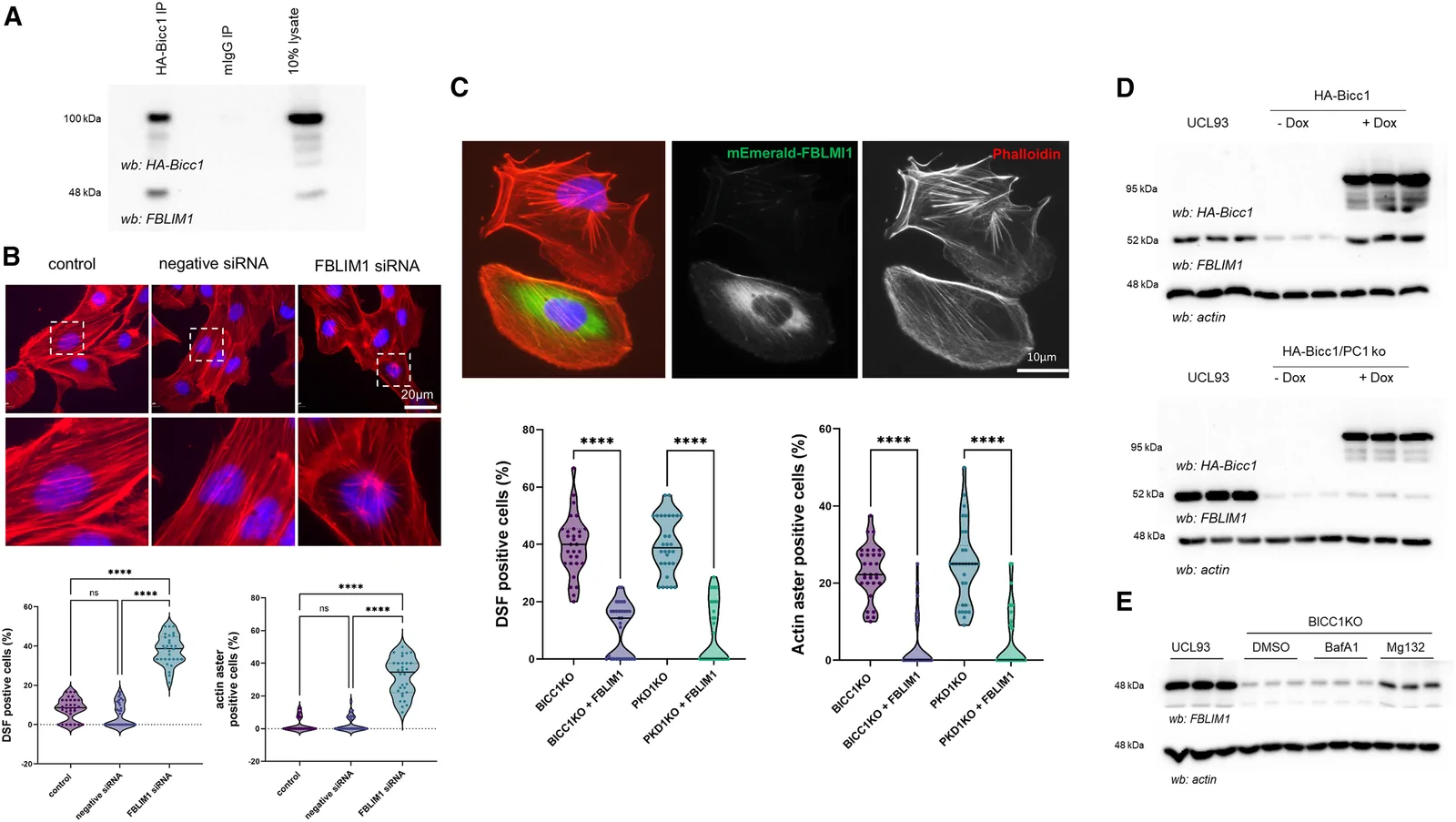
Re-expression of Bicc1 rescues the F-actin phenotype in *BICC1* null cells through inducing FBLIM1 expression (A) CoIP of BICC1 and FBLIM1 demonstrated by HA-pull-down. Non-immune mouse IgG was included as a negative control. Data shown are representative blot of 3 independent experiments. (B) *FBLIM1* siRNA knockdown induced the formation of disordered stress fibers (DSF) and actin asters in UCL93 cells compared with a scrambled siRNA control (*n* = 3 independent experiments, *N* = 30 individual fields). Scale bars, 20 μm. The bottom rows represent an expanded view of the selected windows of the images in the upper rows. One-way ANOVA was performed to assess statistical significance (∗*p* < 0.05, ∗∗*p* < 0.01, ∗∗∗*p* < 0.001, ∗∗∗∗*p* < 0.0001; ns, not significant). siRNA knockdown efficiency of *FBLIM1* indicated by immunoblotting for FBLIM1 and qPCR normalized to Na^+^ K^+^ ATPase (blot) or *ACTB* (qPCR), respectively (not shown). (C) Representative images of *BICC1* and *PKD1* null cells transiently transfected with mEmerald-FBLIM1 and stained with phalloidin to visualize F-actin. Expression of mEmerald-FBLIM1 in transfected cells significantly reduced the formation of disordered stress fibers (DSF) and actin asters in both lines as shown in the violin plots (*n* = 3 independent experiments, *N* = 30 individual fields). Scale bars, 10 μm. One-way ANOVA was performed to assess statistical significance (∗∗*p* < 0.01, ∗∗∗∗*p* < 0.0001). (D) Western blot showing rescue of FBLIM1 expression following induction of HA-Bicc1 expression in *BICC1* KO cells with 1 μg/ml doxycycline for 48 h. FBLIM1 expression was not rescued in *BICC1/PKD1* KO cells. Blots were probed with actin as a loading control. Data shown are representative of 3 repeat experiments. (E) Expression of FBLIM1 was increased in *BICC1* null cells following inhibition of proteasomal (MG132, 10 μM/16 h) but not lysosomal degradation (BafA1, 100 nM/16 h). Blots were probed with actin as a loading control. Data are presented as representative blot of 3 independent experiments.

In wild-type cells, FBLIM1 expression was localized to both FA and intercellular junctions (Figure S10C). We confirmed a biochemical interaction between BICC1 and FBLIM1 proteins by co-immunoprecipitation (coIP) (Figure 6A). Strikingly, newly translated *FBLIM1* (detected by Puro-PLA) was polarized to the basolateral membrane in wild-type cells, and this signal was markedly reduced in both KO cells (Figures S11D and S11E). The overall expression of FBLIM1 protein was also significantly reduced in *BICC1* and *PKD1* null cells. These observations suggest that BICC1 regulates both the translation and stability of *FBLIM1*, contributing to the loss of FBLIM1 protein expression in both null cell types (Figures S10A and S10B).

siRNA knockdown of *FBLIM1* in wild-type cells (UCL93) strikingly phenocopied the actin phenotype observed in *BICC1* null cells (Figure 6B). We hypothesized that FBLIM1 could mediate the actin phenotype observed in *BICC1* and *PKD1* null cells and that re-expression of FBLIM1 could rescue the phenotype in both lines. Indeed, heterologous expression of FBLIM1 in *BICC1* and *PKD1* null cells rescued the actin phenotype, implying a likely functional interaction between all three proteins (Figure 6C).

Of note, HA-Bicc1 re-expression increased FBLIM1 protein expression in *BICC1* null cells but failed to do so in the absence of PC1 i.e., in *BICC1*/*PKD1* null cells (Figure 6D). There was a small but significant increase in *FBLIM1* after BICC1 re-expression (Figure S11C), implying an effect on mRNA stability. Of relevance, FBLIM1 levels were increased in *BICC1* null cells following inhibition of proteasome but not lysosome-dependent degradation indicating that the PC1/BICC1 complex could be protecting FBLIM1 from degradation (Figure 6E). Finally, we compared the expression of FBLIM1 between HEK293-T and UCL93 cells. Expression in the former was 66% lower than in the latter (Figure S12C).

## Discussion

In this study, we identify a BICC1-PC1-ITGB1 signaling module that regulates FA stability and F-actin organization through post-transcriptional, translational, and post-translational control of FBLIM1, thereby maintaining epithelial mechanics and suppressing cyst formation. Disruption of normal F-actin organization is a common feature of cystic kidney cells in *Bicc1* and *Pkd1* mutant mice, but its role in cystogenesis has not been previously studied. F-actin disorganization in null cells was associated with an increase in cellular stiffness but a reduction in traction force, perturbations in mechano-transduction which may further drive changes in gene expression.^27^^,^^28^ We propose that these alterations in cellular mechanics could disrupt tissue integrity to promote cyst formation.

The subcellular localization of endogenous BICC1 had not been previously reported due to the lack of monospecific antibodies. Using newly available tools, we provide clear evidence that BICC1 is widely distributed throughout the kidney epithelial cells, thus expanding present knowledge of its roles. As previously reported for heterologous Bicc1, a fraction was localized to intracellular granules. Previous reports of heterologous BICC1 localization to intracellular RNA granules probably reflect its presence in or around p-bodies^15^^,^^16^ and stress granules,^15^ although the identity of larger aggregates resulting from heterologous BICC1 expression remains unclear.^29^ We confirmed the presence of BICC1 at centrosomes but not in the cilia shaft.^30^^,^^31^

Although the endogenous protein is present in cytoplasmic granules as reported in newborn and adult murine renal tubules,^7^^,^^32^ we observed a novel distinct, granule-independent population of BICC1 that co-localizes with ITGB1 and PXN at the plasma membrane and leading-edge structures. The relative distribution of BICC1 between compartments may be influenced by cell-type-specific regulatory factors such as ANKS3, which is known to disperse BICC1 gel-like polymers,^32^ potentially facilitating the shift of BICC1 toward the membrane-anchored pool.

A plasma membrane-associated BICC1 population has not been previously reported and implies that BICC1’s subcellular distribution is highly dynamic and sensitive to the cellular microenvironment. Indeed, the surface localization of BICC1 is dependent on ITGB1 expression, which may be upregulated in response to the specific mechanical demands of the tubular epithelium or in states of injury. A previous study had implicated ITGB1 as an essential molecule driving cyst formation in a *Pkd1* mouse model.^33^ Our data are consistent with the possibility that ITGB1-regulated BICC1 surface localization at FAs is part of a response to injury program that is also activated under conditions favoring rapid cyst growth to maintain cytoskeletal tension and FA turnover. In this context, the ability of FBLIM1 to activate ITGB1 may also be relevant (see the following text).

PC1 has been previously localized to FA and cell-cell junctions in epithelial cells,^34^^,^^35^^,^^36^ and significant enrichment of AAP in two MS studies of endogenous epitope-tagged murine PC1 has been reported, confirming a physiological role of PC1 at cell adhesion sites and in actin regulation.^27^^,^^37^ Indeed, FA number, stability, composition, and function were all significantly altered in *BICC1* and *PKD1* null cells, suggesting the likelihood that both proteins have a cooperative function in cell adhesion. Our data suggest that PC1 is not required to anchor BICC1 to the plasma membrane. Instead, it appears to stabilize both BICC1 and FBLIM1 protein expression. Conversely, BICC1 interacts not just with ITGB1, but other AAPs localized to FA or involved in actin organization and dynamics confirming a functional link to FA.

Given our recent work identifying BICC1 as a binding partner for both PC1 and PC2,^6^ we investigated whether these cytoskeletal defects were shared by the PC1-PC2 complex or were PC1-specific. However, phalloidin staining revealed that F-actin morphology in PC2 KO cells was indistinguishable from control cells (Figures S12A and S12B). These findings suggest a model where PC1 acts to stabilize the BICC1-FBLIM1 module, a function which does not require PC2-mediated channel activity.

We also observed a striking abnormality in F-actin organization which had been previously observed but its mechanism not clarified. Knockdown of *Bicc1* resulted in prominent stress fiber formation and loss of normal cortical F-actin in mouse IMCD3 cells.^38^ Abnormal proximal tubule F-actin staining had been noted in *Bicc1* KO kidneys at birth, coincident with the onset of cyst formation.^11^ Our results indicate that this is primarily mediated by FBLIM1. Not only did *FBLIM1* knockdown reproduce the actin phenotype seen but heterologous re-expression of FBLIM1 completely rescued the phenotype in *BICC1* and *PKD1* null cells. FBLIM1 was first identified as an interacting partner of FERMT2 and filamin, a scaffold protein associating with F-actin and localizing to cell-matrix adhesions^21^^,^^39^ and cell adherens junctions in an E-cadherin-dependent manner.^40^ *FBLIM1* knockdown has been associated with defects in actin organization and cell-cell junction formation in HT-1080 fibrosarcoma cells.^40^ It may act as a molecular switch for ITGB1 activation by displacing filamin in favor of talin.^41^ *Fblim1* null keratinocytes and bone marrow stromal cells have a common migration defect although no kidney phenotype has been reported so far.^42^^,^^43^

To date, genome-wide analysis of BICC1-regulated target mRNAs has only been conducted in *Drosophila* oocytes and *Xenopus laevis* embryos.^25^^,^^26^ A limited number of mRNAs, notably *PKD2*, have been reported to be regulated by BICC1 in mammalian cells,^11^^,^^15^ but the interactions with *BICC1*, *PKD1*, *FBLIM1*, and *TNS1* mRNAs are novel and imply translational regulation at the global and/or local (FA) level. RNA sequencing in *BICC1* and *PKD1* null cells revealed global changes and commonly altered gene sets. Future studies will seek to refine the set of BICC1-regulated mRNA targets at the genome level and particularly how this might intersect with microRNA (miRNA) regulation especially where relevant to ADPKD pathogenesis.^7^

Disease associations between *BICC1* variants and non-renal phenotypes from genome-wide association studies (GWASs) indicate presently unknown functions outside the kidney.^44^^,^^45^^,^^46^ Recent human population studies have also revealed an unexpected role for heterozygous *BICC1* variants in the incidence and progression of chronic kidney disease (CKD) and kidney failure.^47^^,^^48^ How *BICC1* haploinsufficiency acts as a risk factor for progressive CKD and other non-renal phenotypes merits further investigation, but could relate to it's functions in regulating F-actin and cell adhesion reported here.

**Figure 7:**
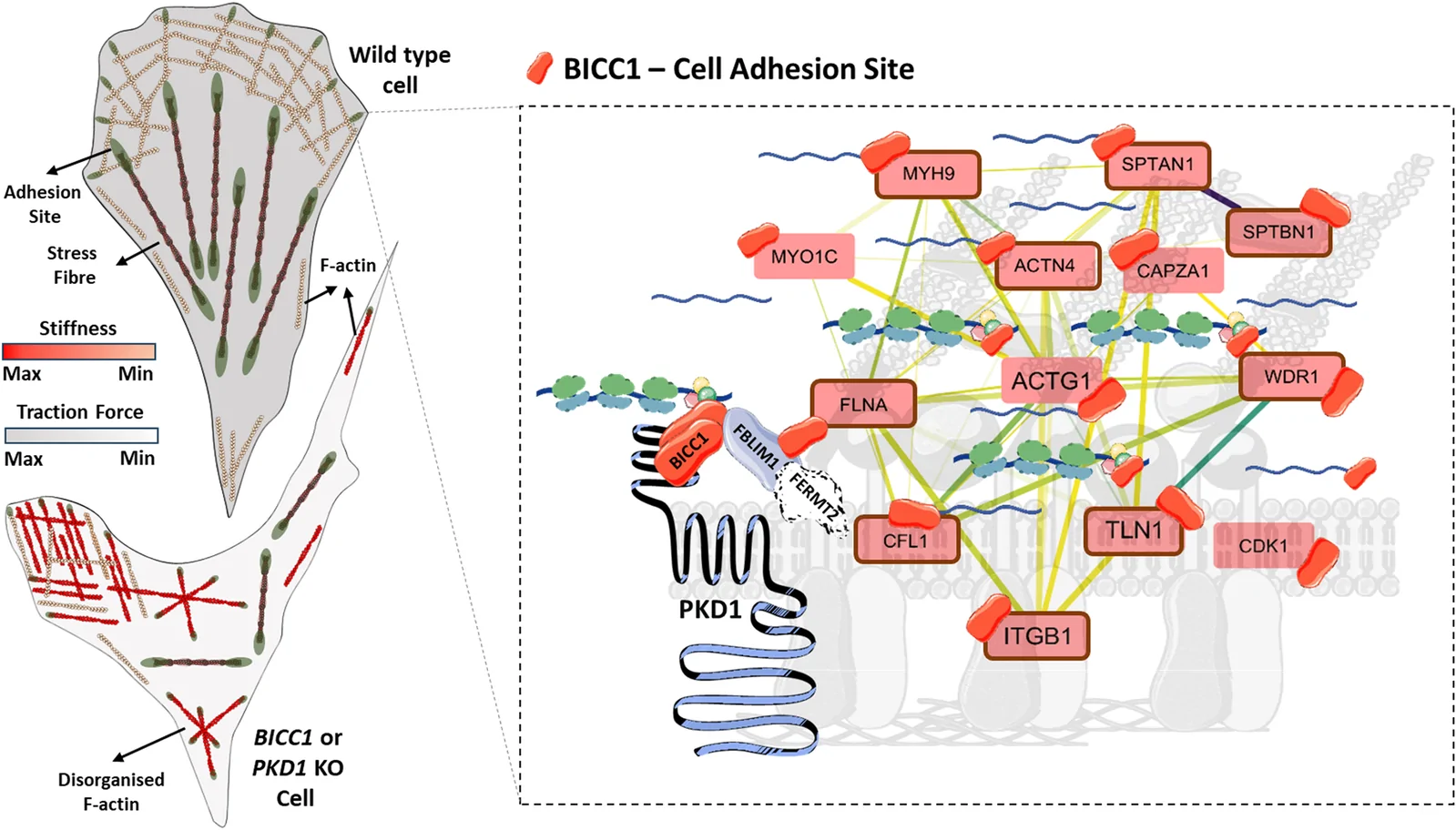
A schematic model of BICC1 and its interactions with key proteins at FA and in F-actin stress fiber organization Normal F-actin stress fiber organization and FA sites in a wild-type cell compared with their disruption in *BICC1* and *PKD1* KO cells. A BICC1-PC1-ITGB1-related protein network at FAs is shown in an expanded view. These likely regulate FA stability, composition, and F-actin stress fiber organization as shown for FBLIM1 in this study. Other FA proteins identified as novel RNA-binding proteins (RBP) in a recent kidney eRIC study^49^ are highlighted with a thicker border. Blue wavy lines represent mRNA associated with or without RBPs.

In summary, we propose a cilia-independent model where BICC1 regulates kidney health through the dynamic regulation of FA structure and function, including through local translation (Figure 7). RNA and RNA-binding proteins have been detected at FA sites in other cell types,^50^^,^^51^ and intriguingly, BICC1 and several of the interacting FA proteins identified in this study have been shown to be RNA-binding proteins.^49^ We conclude that disruption of this intricate network through *BICC1* or *PKD1* mutations results in cyst formation.

### Limitations of the study

The main cellular model studied was a conditionally immortalized human renal proximal tubular cell line. We did not examine cells derived from other tubular segments. The potential functions of BICC1 in other subcellular compartments were not studied.

## Resource availability

### Lead contact

Further information and requests for resources and reagents should be directed to and will be fulfilled by the lead contact, Albert C.M. Ong (a.ong@sheffield.ac.uk).

### Materials availability

Reagents generated in this study will be made available on request, but we may require a completed materials transfer agreement if there is potential for commercial application.

### Data and code availability

• All data supporting the findings of this study are found within the article and its supplemental information. The data generated and used in this study are publicly available with accession numbers documented in the key resources table.
• This article does not report original code.
• Any additional information required to reanalyze the data reported in this article is available from the lead contact upon request.

## Acknowledgments

We thank Oliver Wessely for critical comments on the manuscript, Daniel Constam for the gift of Bicc1 mouse kidney sections, Fiona Wright and Carl Wright for technical assistance. This work was supported by grants from Kidney Research UK and the PKD Charity UK (PKD_RP_005_20211124), the Sheffield Hospitals Charity (Sheffield Kidney Research Foundation) to A.J.S. and A.C.M.O., and the Biotechnology and Biological Sciences Research Council (BB/X008460/1) to C.P.T. D.S. was supported by a Faculty PhD Scholarship from the University of Sheffield. We acknowledge the Henry Royce Institute for Advanced Materials, funded through UKRI-EPSRC (EP/R00661X/1, EP/S019367/1, EP/P02470X/1, and EP/P025285/1) for access to AFM.

## Author contributions

A.J.S., M.K.V., D.S., and L.C. performed the experiments and generated and analyzed the data; I.Z.B.-D., generated and provided key reagents; S.A.W., C.P.T., and A.C.M.O. designed and supervised the experiments; A.C.M.O. conceptualized the project, obtained funding, and wrote the paper. All authors have read and approved the final manuscript.

## Declaration of interests

The authors declare no competing interests.

## STAR★Methods

### Key resources table

**Table:** 

REAGENT or RESOURCE	SOURCE	IDENTIFIER
**Antibodies**		

Rabbit polyclonal anti-BICC1	Sigma-Aldrich	Cat# HPA045212
Mouse monoclonal anti-BICC1	Santa Cruz	Cat# sc-514846
Mouse monoclonal anti-PC1 mAb (Clone 7e12)	Ong et al.^52^	N/A
Rabbit polyclonal anti-PC1 (Clone 2b7)	Newby et al.^36^	N/A
Goat Polyclonal anti-PC2	Santa Cruz	Cat# sc-10376
Mouse ACTB	Abcam	Cat# ab6276
Rat monoclonal anti-HA High Affinity (clone 3F10)	Roche	clone 3F10
Mouse monoclonal anti-GST	Santa Cruz	Cat# sc-138
Rat monoclonal anti-*c*-Myc (clone JAC6)	Biorad	clone JAC6
Rabbit polyclonal anti-GFP	Abcam	Cat# ab6556
Goat anti-mouse IgG	Southern Biotech	Cat# 1030-05
Goat anti-rabbit IgG	Southern Biotech	Cat# 4050-01
Goat anti-rat IgG	Southern Biotech	Cat# 3050-01
Rabbit anti-goat IgG	Dako	Cat# P0449
Mouse monoclonal anti-ARL13B	Proteintech	Cat# 66739-1-Ig
Mouse monoclonal anti-Pericentrin	Abcam	Cat# ab28144
Rabbit polyclonal anti-Pericentrin	Abcam	Cat# ab4448
Rabbit monoclonal anti-CTNNB1	Cell Signaling Tech	Cat# 8480P
Rabbit polyclonal anti-VCL	Proteintech	Cat# 26520-1-A
Mouse monoclonal anti-PXN	BD Biosciences	Cat# 610051
Rabbit polyclonal anti-PXN	Proteintech	Cat# 10029-1-Ig
Rat monoclonal anti-ITGB1 (clone 9EG7)	BD Biosciences	Cat# 553715
Mouse TLN1 8D4 mAb	Abcam	Cat# ab157808
Rabbit polyclonal anti-TNS1	NovusBio	Cat# NBP1-84129
Mouse monoclonal anti-FBLIM1	Santa Cruz	Cat# sc-271417
Rabbit polyclonal FBLIM1	Proteintech	Cat# 13349-1-AP
Mouse monoclonal anti-Puromycin (clone 12D10)	Sigma-Aldrich	Cat# MABE343
Texas Red™-X Phalloidin	Invitrogen	Cat# T7471
Wheat Germ Agglutinin (WGA)-AF488	Invitrogen	Cat# W11261
Duolink Probe Anti-Rabbit PLUS	Merk	Cat# DUO92002
Duolink Probe Anti-Mouse MINUS	Merk	Cat# DUO92004

**Biological samples**		

pSpCas9(BB)-2A-Puro (PX459) V2.0	Addgene	plasmid #62988
pSpCas9n(BB)-2A-Puro (PX462) V2.0	Addgene	plasmid #62988
mEmerald-Migfilin-C-14	Addgene	plasmid # 54181
pFRT/TO/FLAG/HA-DEST	Invitrogen	N/A
pcW57.1	Addgene	plasmid #41393
pcW57.1-HA-Bicc1	This Study	N/A

**Chemicals, peptides, and recombinant proteins**		

Duolink Reagents	Merck	DUO92008

**Critical commercial assays**		

Plasma membrane protein extraction kit	Abcam	ab65400
Matrigen SoftTrac™ 35 mm dishes	Cellguidance	SV3520-COL-1
MISSION Lentiviral Packaging Mix	Sigma	SHP001
ITGB1 siRNAs SmartPool	Dharmacon	L-004506-00-0005

**Deposited data**		

Transcriptome Profiling of UCL93 Kidney Epithelial Cells and CRISPR-Cas9 Knockouts for PKD1 and BICC1 Using Total RNA-Seq	ArrayExpress https://www.ebi.ac.uk/biostudies/ArrayExpress/	E-MTAB-14624
Original Western blots from figures	Mendeley	https://doi.org/10.17632/rzd4d42xbt.1

**Experimental models: cell lines**		

UCL93	Streets et al.^35^	NA
UCL93 PC1 KO c1	This study	NA
UCL93 PC1 KO c7	This study	NA
UCL93 PC1 KO c11	This study	NA
UCL93 PC1 KO c17	This study	NA
UCL93 PC1 KO c24	This study	NA
UCL93 BICC1 KO c3	This study	NA
UCL93 BICC1 KO c4	This study	NA
UCL93 BICC1 KO c20	This study	NA
UCL93 BICC1 KO c35	This study	NA
UCL93 BICC1 KO c36	This study	NA
UCL93 BICC1 KO c40	This study	NA
UCL93 PKD2 KO c20	This study	NA
Flp-In T-REx HEK293 cells	Invitrogen	
HA-BICC1 HEK293-TRex cells	This study	NA

**Experimental models: organisms/strains**		

Pkd1 mice (Pax8rtTA-TetO-Cre-Pkd1fl/fl) PN23	This study	N/A
Bicc1^−/−^ kidney tissue sections (E18.5)	Provided by D Constam	N/A

**Oligonucleotides**		

*PKD1* Forward: *TCTGAGGAACCTGAGCCCTA, Reverse: AGTGGCTGGAGAGGTTCAGA*	IDT	N/A
*PKD2* Forward: *AGCCTGGATGACTCTGAGGA, Reverse: TGGCTCGCTCCATAATCTCT*	IDT	N/A
*BICC1* Forward: *AACAGCCAAGCAAGTCTGTG, Reverse: ACTGCTTTCAAGTCCGAGGA*	IDT	N/A
*FBLIM1 Forward: CGGCAGAACCTGTTGAGAAAGG, Reverse: ACGTGAAGCACTGGGCATGGTA*	IDT	N/A
*TNS1 Forward: ACTCCAGAGGAGGAGCCATTGA, Reverse: TGTGGCTTCTGGAGACTGGTTC*	IDT	N/A
*ACTB Forward: AGGATTCCTATGTGGGCGAC, Reverse: ATAGCACAGCCTGGATAGCAA*	IDT	N/A

**Software and algorithms**		

CZ CELLxGENE Discover	https://github.com/chanzuckerberg/single-cell-data-portal	N/A
EzColocalization	https://github.com/DrHanLim/EzColocalization	N/A
Serialcellpose	https://www.napari-hub.org/plugins/napari-serialcellpose	N/A
Cellpose	https://github.com/BioImaging-NKI/Cellpose-Fiji	N/A
StarDist	https://imagej.net/plugins/stardist	N/A
ImageJ/Fiji	https://imagej.net/	N/A
Napari	https://napari.org/	N/A
Intervene	https://github.com/asntech/intervene	N/A
Bioicons	https://github.com/duerrsimon/bioicons	N/A
STRING v12	https://string-db.org/	N/A
morpheus.R	https://github.com/cmap/morpheus.R	N/A
VolcaNoseR	https://github.com/JoachimGoedhart/VolcaNoseR	N/A
MotilityLab/celltrackR	https://github.com/ingewortel/celltrackR	N/A
TrackMate	https://imagej.net/plugins/trackmate/	N/A
AGAVE v1.1.0	https://github.com/allen-cell-animated/agave	N/A
Directionality	https://imagej.net/plugins/directionality	N/A
ZEISS ZEN 3.0	ZEISS	N/A
SimplePCI	Compix	N/A
WebGestaltR	https://github.com/bzhanglab/WebGestaltR	N/A
Sheffield Advanced Research Computer (ShARC)	University of Sheffield	N/A
star = 2.6.1	https://github.com/alexdobin/STAR	N/A
rsem = 1.2.28	https://deweylab.github.io/RSEM/	N/A
fastqc-0.11.8-1	https://www.bioinformatics.babraham.ac.uk/projects/fastqc/	N/A
DEGandMore	https://github.com/zhezhangsh/DEGandMore	N/A
Image Lab 5.1	Bio-Rad	N/A
RStudio	https://rstudio.com/	N/A

**Other**		

Bruker Bioscope Resolve - Nikon Eclipse Ti2	Bruker - Nikon	Royce, UOS
ZEISS Celldiscoverer7	ZEISS	LMF, UOS
ZEISS LSM 980 with Airyscan 2	ZEISS	LMF, UOS
Olympus Inverted IX71	Olympus	LMF, UOS
QuantStudio 7	Life Technologies	UOS
Bio-Rad ChemiDoc XRS+	Bio-Rad	UOS

### Experimental model and study participant details

#### Cell culture

All cell lines used in this study were routinely tested for mycoplasma contamination every two months using EZ-PCR Mycoplasma Test Kit (Geneflow, UK) and were confirmed negative prior to and during experimentation.

UCL93 kidney epithelial cells were immortalized from primary cultures of tubular cells isolated from normal human kidneys removed for clinical indications as previously described.^35^ Cells were grown in Dulbecco’s modified Eagle’s medium-Ham’s 12 (DMEM-F12, Invitrogen) supplemented with 1% L-glutamine (Invitrogen), 5% NuSerum (Becton Dickinson), and 1% antibiotic/antimycotic solution (Invitrogen) at 33°C/5% CO2. HEK-293T cells were cultured in Dulbecco’s modified Eagle’s medium-Ham’s 12 (DMEM-F12, Invitrogen) supplemented with 1% l-glutamine (Invitrogen), 10% FCS, and 1% antibiotic/antimycotic solution (Invitrogen) at 37°C/5% CO2.

#### Generation and validation of FLAG/HA-tagged BICC1 HEK293 inducible cell lines

To generate stable HEK293 cell lines that inducibly express FLAG/HA-tagged BICC1 isoforms 1 and 2, the Flp-In T-REx system and Gateway recombination cloning technology were utilized.^53^^,^^54^ In brief, human BICC1 coding sequences were PCR-amplified from human podocyte cell line cDNA and cloned into a pENTR4 Gateway entry vector containing attL sites (Invitrogen). Two distinct inserts corresponding to isoforms 1 (excluding exon 21) and 2 (including exon 21), were identified by Sanger sequencing as described in mice^4^ and confirmed by targeted PCR in ADPKD patient urine cDNA (not shown). pENTR4 BICC1 isoform 1 and pENTR4 BICC1 isoform 2 were then recombined into pFRT/TO/FLAG/HA-DEST destination vectors using GATEWAY LR recombinase to allow for doxycycline-inducible expression of stably transfected FLAG/HA-tagged protein from the TO/CMV promoter. Flp-In T-REx HEK293 cells (Invitrogen) were transfected with the expression constructs and pOG44 encoding the Flp recombinase in a 1:9 ratio then subjected to selection with hygromycin and blasticidin (to maintain TetR expression) to generate stable cell lines. Doxycyline-inducible expression of BICC1-isoform 1 and isoform 2 was confirmed by Western blotting with an anti-HA antibody (Covance Cat# MMS-101P) and anti-BICC1 mAb (2D9, Sigma-Aldrich Cat# MABE344).

#### CRISPR/CAS9 mutagenesis

UCL93 cells were transfected with pSpCas9(BB)-2A-Puro (PX459) V2.0 (pSpCas9n(BB)-2A-Puro (PX462) V2.0 (Addgene plasmid #62988, kind gift of Feng Zhang) containing a gDNA targeting the first exon of *PKD1* (5′-CACCGCGCCGGGCGCTGGGCCGCAG) or the first exon of *BICC1* (5′-CACCGGGAGAGCCCGGCTACCTGG). Positive clones were selected for puromycin resistance followed by limiting dilution. Mutations were then validated by genomic DNA sequencing and western blotting using specific antibodies to PC1 (7e12) and BICC1 (Sigma-Aldrich Cat# HPA045212). The generation of *PKD1* null cells has been previously reported.^8^

#### Lentiviral rescue of Bicc1

N-terminal HA epitope tagged Bicc1 was cloned into pcW57.1 (Addgene plasmid #41393, kind gift of David Root). Viral particles were generated from HEK-293T cells following co-transfection of pcW57.1-HA-Bicc1 and MISSION Lentiviral Packaging Mix (Sigma, UK). Following incubation, stably transduced UCL93 BICC1 knockout cells were selected for puromycin resistance followed by limiting dilution. Inducible expression of HA-Bicc1 in *BICC1* and *BICC1/PKD1* knockout cells was initiated following incubation with 1 μg/ml doxycycline (Sigma, UK) for 24 or 48h. BICC1 point mutations (human reference) were generated in HA-Bicc1 by site-directed mutagenesis as previously described.^55^

#### *In vivo* experiments

All experimental protocols involving animals were reviewed and approved by the Ethical Review Board of the School of Medicine and Population Health at the University of Sheffield and were conducted in strict accordance with UK national guidelines under Home Office Project Licence PP9820851.To account for sex as a biological variable, kidney tissue was harvested from equal numbers of mice (*n* = 3 males, *n* = 3 females). Animals were humanely euthanized via Schedule 1 cervical dislocation prior to tissue harvest. Immediately following collection, kidney tissues were snap-frozen in liquid nitrogen and stored at −80C prior to analysis.

*Pkd1* deletion was induced by doxycycline injections at postnatal days (PNs) 13–15 in tetracycline-inducible, kidney-specific *Pkd1* mice (*Pax8rtTA-TetO-Cre-Pkd1fl/fl*). After sacrifice at PN23, *Pkd1* ko and WT control kidneys were collected and embedded in cry-*M*-bed solution (Wolflabs) or immersed in 10% neutral buffered formalin (Millipore) for histological analysis. Tissue sections were processed for immunofluorescence staining. *Bicc1*^−/−^ kidney tissue sections (E18.5) were the kind gift of Prof Daniel Constam (École Polytechnique Fédérale de Lausanne, Switzerland).

### Method details

#### Materials

All chemicals were purchased from Sigma Chemical (Poole, Dorset, United Kingdom), unless otherwise stated.

#### Antibodies

For IPs and western blotting, Primary antibodies used in this study were rabbit BICC1 (Sigma-Aldrich Cat# HPA045212), mouse BICC1 mAb (Santa Cruz Cat# sc-514846), PC1 mAb (7e12),^52^ rabbit PC1 (2b7),^36^ goat PC2 (Santa Cruz Cat# sc-10376), mouse ACTB (Abcam Cat# ab6276), HA High Affinity (rat monoclonal clone 3F10, Roche), mouse GST (Santa Cruz Cat# sc-138), rat c-Myc (clone JAC6, Biorad) and rabbit GFP (Cat# ab6556, Abcam). All primary antibodies were used at 1:1000 unless otherwise stated. Secondary antibodies used in this study include goat anti-mouse IgG (Cat# 1030-05, Southern Biotech), goat anti-rabbit IgG (Cat# 4050-01, Southern Biotech), goat anti-rat IgG (Cat# 3050-01, Southern Biotech) and rabbit anti-goat IgG (Cat# P0449, Dako). All secondary antibodies were used at 1:10,000, unless otherwise stated in the results section.

For staining, primary antibodies used were specific to rat HA High Affinity (1:300), rabbit BICC1 (1:200), mouse ARL13B (1:250, 66739-1-Ig, Proteintech), mouse/rabbit Pericentrin (1:250, ab28144 and ab4448, Abcam), rabbit CTNNB1 (1:250, 8480P Cell Signaling Tech), rabbit VCL (1:500, 26520-1-AP, Proteintech), mouse/rabbit PXN (1:500, 10029-1-Ig, Proteintech and 610051, BD Biosciences), rat ITGB1 (1:100, 9EG7 553715, BD Biosciences), mouse TLN1 (1:500, 8D4 ab157808, Abcam), rabbit TNS1 (1:250, NBP1-84129, NovusBio) and mouse/rabbit FBLIM1 (1:250, SC-271417, Santa Cruz Biotechnology and 13349-1-AP, Proteintech). Secondary antibodies AF488 or AF594 for either goat anti rabbit or mouse or rat (IgG (H + L) Highly Cross-Adsorbed Secondary Antibody, Alexa Fluor™, Invitrogen). All secondary antibodies were used at 1:500. Conjugates used in this study are Texas Red™-X Phalloidin (1:1000, T7471, Invitrogen) and Wheat Germ Agglutinin (WGA)-AF488 (1:1000, W11261, Invitrogen).

#### Attachment, detachment and spreading assays

For attachment and spreading assays, cells were passaged and 10000 cells/well allowed to attach for 30 min. Unattached cells were washed away with PBS and remaining cells counted and cell area calculated using ImageJ. For cell detachment assay cells were plated in 96 well plates at 1000 cells/well for 48h. Cells were incubated with 1 mM EDTA/PBS for 30 min and attached cells stained with 0.02% crystal violet for 30 min. Excess dye was discarded by washing the plates for 15 min in fresh water. The cell-bound dye was redissolved in 70% (v/v) ethanol/water and the optical density was measured at λ = 570 nm.

#### Matrigel 3D cyst assays

3D Matrigel cyst assays were performed as previously described.^13^ In brief, UCL93 cells (1 × 10^5^/well) were mixed with 70 μL Matrigel (Becton Dickinson, UK), plated into 96 well plates in triplicate and incubated for 30 min at 37°C to facilitate gel formation. Cells were then cultured for 12days in the presence of vehicle (DMSO) or 50 μM CK-666 (Tocris, UK). Media was replaced every 2 days. The percentage of tubular structures was calculated from 3 independent wells per treatment.

#### Transfections

Cells were transfected using Lipofectamine 3000 (Life Technologies) or Amaxa electroporation (program W-01) for 48 h before the cell assays. For siRNA knockdown assays, cells were transfected with negative control or specific ITGB1 siRNAs (SmartPool, Dharmacon, USA) using RNAimax (Life Technologies). siRNA knockdown was confirmed by qPCR using specific Taqman probes. mEmerald-Migfilin-C-14 was a kind gift from Michael Davidson (Addgene plasmid # 54181).

#### RT-qPCR

Relative mRNA expression was determined by SYBR green qPCR according to the manufacturer’s protocol (Life Technologies). Following RNA extraction, cDNA was synthesised using a total RNA to cDNA kit (Life Technologies). Real-time PCR was carried out on a QuantStudio 7 qPCR machine. Normalisation of expression was carried out using specific primers to actin. Relative quantification carried out using the comparative Ct (ΔΔCt) method to calculate fold changes in expression between samples.

#### Protein biochemistry

Cells were lysed by extraction at 4°C using the IP lysis Buffer (25 mM NaCl, 150 mM EDTA, 1 mM 0.5% NP40, 1% Triton X-100, pH 7.0) supplemented with a protease inhibitor cocktail (Roche). Immunoblotting and immunoprecipitation were performed as previously described.^36^ To identify proteins bound to actin pulldown was carried out using biotin-XX phallodin (Thermo-Fisher Scientific, UK) as described.^56^ Briefly cell lysates were incubated overnight with 2 mM biotin-XX phalloidin followed by capture with avidin Dynabeads C1 for 30 min. Lysates were washed with IP lysis buffer, 500 mM NaCl and bound proteins detected by Western blot.

Due to concerns regarding antibody specificity and sensitivity, we employed membrane fractionation to confirm both plasma membrane and cytoplasmic components of endogenous BICC1. A plasma membrane protein extraction kit (Abcam, UK ab65400) was used to isolate plasma membrane and cytosolic protein fractions according to the manufacturer’s instructions. For protein degradation assays, cells were incubated with 10 μM Mg132 or 100 nM BafA1 for 16h. Bio-Rad ChemiDoc XRS+ and Image Lab 5.1 software were used for visualization and quantification of proteins of interest. All quantification was carried out on non-saturated bands as determined by the software from 3 independent experiments.

#### LC-MS/MS analysis

During the sample preparation phase, HEK293 (Control) and HA-Bicc1 cells were plated at 1 × 10^5^ cells/mL and grown to 90% confluency. BICC1 and its protein binding partners were immunoprecipitated from cell lysates using anti-HA antibody beads. To test the efficiency of the Co-IP, a Western blot experiment was performed during the standardization phase. LC-MS was done at the Fingerprints Proteomics department at the University of Dundee. Peptides were extracted from 1D SDS-PAGE gel slices and subjected to tryptic digestion. The trypsinised digests of each gel section were run on a 1D NanoLC ESI MS/MS instrument. The sample peptides are separated within the Capillary LC system, using a reverse phase C18 column. The peptides are then eluted directly into the MS/MS instrument and detected as ions in the MS mode or further fragmented and detected in MS/MS mode. Samples were analyzed using Mascot (Matrix Science, Boston, MA, USA), protein scores from HA-Bicc1 (*n* = 3) and control (*n* = 3) were merged and missing values were imputed by fitting a minimal value to a normal distribution. Further merged data was quantile normalised for differential enrichment analysis using R package DEGandMore (NormQQ and StudentsT Method, https://github.com/zhezhangsh/DEGandMore), the *p*-value of <0.05 being considered significant. Significantly enriched candidates were then classified as high-confidence interacting proteins (HCIPs) or non-specific binders in combination with the CRAPome contaminant database.^57^

#### Total RNA sequencing (RNA-Seq) and analysis

UCL93 and KO cells were plated at 5 × 10^5^ cells/mL in 10 cm^2^ plates and incubated at 33°C until 70% confluency was reached. Total RNA was extracted from the cells (*n* = 3/cell line) using a Trizol reagent. After rRNA removal, the NGS libraries were constructed and sequenced (Paired-end 150 bp) at the Novogene facility. Data was processed in ShARC (Sheffield Advanced Research Computer at the University of Sheffield). Quality control checks on raw sequence data were done using the bioconda package fastqc-0.11.8-1. The adapter trimming was performed using cutadapt-1.18, and reads were aligned to the human genome (human genome GRCh38 and annotations gencode.v29) using star = 2.6.1 and Transcriptome.out.bam was realigned and quantified using rsem = 1.2.28. Expected counts and TPM values were extracted from gene-level expression estimates files for further analysis. The expected counts were subjected to DESeq2 normalisation and two-group differential expression analysis using the R package DEGandMore, the *p*-value of <0.05 being considered significant. Gene Set Enrichment Analysis (GSEA) was conducted using the toolkit WebGestaltR, by selecting functional databases Geneontology, KEGG and Reactome.

#### Immunofluorescence and imaging

Semi-confluent UCL93 and KO cells on coverslips or plates were fixed with 4% paraformaldehyde or 10% Formalin in DPBS for 10 min. Cells were then washed and permeabilized in 0.5% Triton X-100 in DPBS with calcium and magnesium for 5–15 min. Cells were blocked with 3% BSA for 1 h, incubated for either 1 h at room temperature or overnight at 4°C with primary antibodies, and incubated for 1 h with secondary antibodies. After washes, coverslips were mounted using ProLong™ with NucBlue™ Stain and imaged on a ZEISS Celldiscoverer7 Widefield Fluorescence Microscope, ZEISS LSM 980 with Airyscan 2 and Olympus Inverted IX71 fluorescence microscope (Light Microscope Facility, The University of Sheffield). For Airyscan imaging, 405, 488 and 561 nm laser lines were used and fixed cells were imaged with a Plan-Apochromat 63×/1.4 numerical aperture (NA) oil objective on a ZEISS LSM 980 with Airyscan 2. For all fixed-cell imaging, images shown are snap images and maximum intensity z projections, unless otherwise noted. Images were acquired using ZEISS ZEN Microscopy Software and SimplePCI imaging software (Compix, Hamamatsu, PA). Airyscan images were further processed in ZEISS ZEN 3.0 blue edition software.

#### Actin directionality, proportional analysis and thickness

To investigate changes in directional orientation of actin stress fibers, Fourier component analysis for directionality (Goodness of Gaussian fit and angular dispersion of the actin stress fibers) was performed on F-actin channel ROI single cell images using the ImageJ plug-in ‘Directionality’ created by Jean-Yves Tinevez (https://imagej.net/plugins/directionality) following their respective instructions. Numerical values of the stress fiber direction, angular dispersion and goodness were exported to an Excel spreadsheet for further analysis. The main actin phenotype was quantified as the proportion of cells with disorganised stress fibers (DSFs) with higher angular dispersion and poor Gaussian fit or the presence of actin asters. Pathtrace view of actin organisation was generated using AGAVE v1.1.0 (https://github.com/allen-cell-animated/agave). The thickness of the actin cortex, measured in micrometers (μm), was determined from images of Phalloidin-stained cells. This measurement was performed manually by drawing a line across the actin cortex using the line profile tool in Zen Blue 3.0.

#### Cilia quantification and proportion analysis

For cilia length measurements, ImageJ analysis software (NIH) was used to measure >100 cilia in at least 3 independent experiments. For proportion analysis, images of cells were collected using 10× magnification on a ZEISS Celldiscoverer7 Widefield Fluorescence Microscope and captured images were pre-processed for noise reduction and cilia segmentation. Next, particle Analysis was employed to identify individual cilia based on their size and shape (circularity <0.5). Converted masks were used to quantify the number of cilia in ROIs and % cells with single cilium. To investigate changes in directional orientation of cilia, Fourier component analysis for directionality was performed on ARL13B channel ROI images using the ImageJ plug-in ‘Directionality’ following their respective instructions. Numerical values of the cilia main direction, angular dispersion and goodness were exported to an Excel spreadsheet for further analysis.

#### Atomic force microscopy (AFM)

AFM measurements were performed with a Bruker Bioscope Resolve mounted on an inverted microscope (Nikon Eclipse Ti2) connected to an ORCA-Flash4.0LT (Hamamatsu) camera. Cells were seeded on 50 mm cell culture dishes (Willco GWST-5040) and allowed to adhere at 20% confluency for imaging. Imaging was performed in filtered PBS at room temperature. Pre-calibrated Silicon Tip – Nitride cantilevers (PFQNM-LC-V2 Bruker) were used with a 70 nm tip radius. PeakForce QNM/PeakForce Tapping with a maximum indentation force of 0.5 nN was used. Indentation curves were fitted within Nanoscope Analysis (Bruker) using a cone-sphere model^58^^,^^59^ and an *R*^2^ selection of >0.7. 10 regions of interest were selected for each cell which represents a total of 10,000 force curves per cell to calculate the Young’s Modulus.

#### Traction force microscopy

Traction force microscopy (TFM) experiments were performed as previously described.^60^ Traction force microscopy experiments were performed using collagen-coated 1 kPa hydrogels with 0.2 μm red fluorospheres Matrigen SoftTrac™ 35 mm dishes (Cellguidance-Cambridge). Dishes were equilibrated with cell culture medium for 30 min at 33°C. UCL93 and KO cells were seeded and allowed to adhere overnight at 20% confluence for imaging. Phase images of attached cells and fluorescent embedded beads were collected using 40× magnification on a ZEISS Celldiscoverer7 Widefield Fluorescence Microscope. Images of the fluorescent microspheres on a relaxed hydrogel were collected after 5 min treatment with a 0.5% (w/v) Triton-X-100, 20 mM NH_4_OH PBS solution to remove cells. The displacement of beads before and after the removal of cells was tracked by particle imaging velocimetry followed by Fourier transform traction cytometry to estimate the corresponding cell traction force field.^61^^,^^62^ The total traction forces (in N) were measured by integrating traction forces over the cell area. The elastic energy (in J) stored in the gel to produce the observed deformation was calculated by summing the products of displacement with the force over the cell area.

#### Single cell migration assay

UCL93, and KO cells were seeded and allowed to adhere at 20% confluence (6 h), cells were incubated with NucBlue™ Live ReadyProbes™ Reagent (Hoechst 33342) (added to cell culture), after 1-h cells were washed with PBS and replaced with fresh media for Time-lapse imaging. Phase contrast and Hoechst 33342 images of attached were collected using 20× magnification on a Zeiss Celldiscoverer7 Widefield Fluorescence Microscope for every 12 min up to 12 h (Carl Zeiss Microscopy, Thornwood, NY). Captured images were pre-processed for noise reduction and contrast enhancement. Cell tracking was performed using Fiji/ImageJ software with the TrackMate plugin created by Jean-Yves Tinevez (https://imagej.net/plugins/trackmate/) following their respective instructions. Cell nuclei or phase contrast cell image were identified using segmenter called trackmate-thresholding-detector and TrackMate-clij2. The TrackMate algorithm called Kalman tracker was then employed to track individual cells over time based on their centroids. Trajectories of individual cells were analyzed using TrackMate Analysers. Using optimised parameter file, all the images from three independent replicates were batch processed using the TrackMate Batcher. Trackmate files were merged using inbuilt merge function and exported for MotilityLab/celltrackR directionality analysis (https://github.com/ingewortel/celltrackR). Track data from multiple files was merged for further statistical analysis. Track displacement: Measure the distance between the last spot of the track and the first spot of the track in time. Confinement ratio: how “efficient” was a track displacement in getting far away from its starting point, it is defined as the net-displacement divided by the total-distance, values close to 0 indicate a confined movement, where the particle would stay close to its starting point and values close to 1 indicate that the particle travels along a line with a constant orientation. The net-displacement is given by the Track displacement feature, and the total distance is given by the Total distance traveled feature.

#### Low formaldehyde RNA immunoprecipitation

Formaldehyde RNA immunoprecipitation (fRIP) was carried out as previously described.^63^ Protein G Dynabeads (Novex by Life Technologies) were used to immunoprecipitate endogenous BICC1 with 4 μg of anti-mouse BICC1 mAb (Santa Cruz Biotechnology Inc) or non-immune mIgG. The specificity of the BICC1 antibody for endogenous BICC1 is shown in Figure 1A. The beads/antibody suspension was incubated at room temperature for 1 h before being washed twice with 900 μL lysis buffer. The remaining volume of the cleared lysate (approximately 300 μL) was added to the beads and the sample were rotated at 4°C for 2 h. The samples were washed twice with 900 μL lysis buffer followed by washes with 900 μL high salt lysis buffer (0.5 M NaCl) and then washes with 900 μL normal lysis buffer. Following these washes, 56 μL ice-cold RNase free H_2_O was added to the beads and 26 μL to the inputs, before 33 μL of the 3× reverse-crosslinking buffer (3× PBS, 6% N-lauroylsarcosine, 30 mM EDTA, 15 mM DTT), 10 μL proteinase K (Roche 19 mg/uL) and 1 μL RNase inhibitor was added. The beads were resuspended by flicking the tube gently and were then incubated at 42°C for 1 h followed by 55°C for 1 h with shaking at 1100rpm. The samples were placed on a magnet and the eluates were collected and the total volume was made to 250 μL (addition of approximately 150 μL) with ice-cold RNase free H2O. RNA was isolated and treated with DNase I. RT-qPCR was carried out using specific primers to candidate genes: *PKD1, PKD2, BICC1, FBLIM1, TNS1, ACTB*.

#### Proximity ligation assay (PLA) and NSPs plus PLA (Puro-PLA)

For *In situ* detection of endogenous BICC1 and ITGB1 interactions by PLA, UCL93 and KO cells cultured semi-confluent on coverslips fixed and incubated with mouse anti-BICC1 (1:250) and rabbit anti-ITGB1 (1:250) antibody overnight. For Puro-PLA, cells were incubated with puromycin (10 μg/ml, up to 12 min) for the condition and with cycloheximide (100 μg/ml, up to 3 h) plus puromycin (10 μg/ml, up to 12 min) for the controls, treated cells were fixed and incubated with mouse anti-puromycin (1:500) and rabbit anti-FBLIM1 (1:250) antibodies overnight. Cycloheximide (CHX) is a potent inhibitor of ribosome-mediated translation elongation. It halts the ribosome before puromycin can be incorporated into nascent polypeptide chains, serving as a negative control.^64^ PLA was performed using Duolink Reagents (DUO92008, Merck) following the manufacturer’s instructions.^65^ Secondary antibodies Duolink® *In Situ* PLA® Probe Anti-Rabbit PLUS and Anti-Mouse MINUS (DUO92002 and DUO92004, Merck) were used at 1:5. Cells were incubated with Wheat Germ Agglutinin (WGA)-AF488 (1:1000) for 10 min before the last wash and mounted using ProLong™ with NucBlue™ Stain. Images of cells were collected using 20× and 63× magnification as apical-basal z stack or focused to the basal side of cells on a ZEISS LSM 980 with Airyscan 2.

#### Tissue immunofluorescence

Frozen tissue sections were placed in 4% paraformaldehyde in PBS for 20 min and sections were washed with PBS twice for 5 min. The tissue sections were then permeabilised with 0.1% v/v Tween 20 (Sigma-Aldrich, St. Louis, MO, USA) in PBS for 10 min and washed again with PBS for 5 min. Paraffin embedded kidney sections were dewaxed and rehydrated before antigen unmasking in a boiling citrate solution (1 mmol/L Tri-sodium citrate, pH 6.0, VWR) for 10 min All tissue sections were then blocked with 2% w/v BSA in PBS (incubation buffer) for 20 min. For actin filament staining, the frozen sections were incubated with a 1:20 dilution of phalloidin in incubation buffer for 60 min. After the incubation, the sections were washed three times with PBS, 5 min each. For FA markers paraffin embedded sections were incubated overnight with primary antibodies to FBLIM1 (Santa Cruz) or ITGB1 (Proteintech). After 3 × 5min PBS washes sections were incubated with Alexa Fluor 488 secondary antibodies (Invitrogen, USA) for 1h. After washes coverslips were mounted using ProLong™ with NucBlue™ Stain and imaged on ZEISS LSM 980 with Airyscan 2 (Light Microscope Facility, The University of Sheffield). Airyscan images were further processed in ZEISS ZEN 3.0 blue edition software.

### Quantification and statistical analysis

#### Data visualisation and statistical analysis

Data are presented as mean values ±SEM. Parametric Student’s *t* test and one-way ANOVA were used for statistical analysis with a *p*-value of <0.05 indicating statistical significance (∗*p* ≤ 0.05, ∗∗*p* ≤ 0.01, ∗∗∗*p* ≤ 0.001 and ∗∗∗∗*p* ≤ 0.0001). Plotting and analyses were carried out using Prism 9 (Graphpad). Heat maps were generated using morpheus.R (https://github.com/cmap/morpheus.R) and Volcano plots were created using VolcaNoseR (https://github.com/JoachimGoedhart/VolcaNoseR).

#### Cytoscape network analysis

Protein-protein interaction and gene network analysis was conducted for proteins or genes of interest using a StringApp in Cytoscape v3.9. Network Nodes were colour-coded for differential expression (RNA-seq) or enrichment (MS), edges were colour-coded by co-expression scores and thickness by confident scores from the STRING v12 database. The network was exported to Draw.io and overlaid on a representative cell adhesion image generated using Bioicons seriver CC BY 4.0 (https://github.com/duerrsimon/bioicons).

#### Actin and adhesion proteins (AAPs) data curation and analysis

We curated a list of AAPs from several published datasets (*n* = 496, Figure S6). Three major datasets were an F-actin interactome obtained by phalloidin pull-down,^66^ a *b-Actin* mRNA interactome and integrin adhesion complexes (IACs) by proximity biotinylation and mass spectrometry.^67^^,^^68^ We compared these AAPs with differentially expressed genes detected in the KOs by analysing the relationships between sets and their intersections. UpSet plots were created using intervene (https://github.com/asntech/intervene). A Kidney, Liver and Brain enhanced RNA interactome capture (eRIC) dataset was analyzed to identify potential associations with BICC1 interacting IACs identified in this study.^49^

#### Segmentation and colocalization analysis

Image analysis was conducted using ImageJ/Fiji (https://imagej.net/) and Napari (https://napari.org/). Airyscan-processed focal adhesion images were pre-processed to enhance contrast and reduce noise using background subtraction and Gaussian blurring. To segment focal adhesions, the default dark thresholding method was applied to binarize the image, followed by morphological operations such as erosion and dilation to remove noise and refine object boundaries. Next, particle analysis was employed to identify individual focal adhesions based on their size and shape. Additional features such as circularity (<0.5) and aspect ratio were used to discriminate against non-specific structures. Converted masks were used for further analysis such as to quantify number of FA sites including the intensity of protein expression in segmented FA sites. Nuclear objects were segmented using the ImageJ plug-in ‘StarDist’ (versatile fluorescent nuclei, https://imagej.net/plugins/stardist) following their respective instructions. Cells were segmented using the ImageJ plug-in ‘Cellpose using the BIOP-EPFL’ (https://github.com/BioImaging-NKI/Cellpose-Fiji) or napari plug-in ‘serialcellpose’ (https://www.napari-hub.org/plugins/napari-serialcellpose) following their respective instructions. Colocalization analysis was conducted using the ImageJ plug-in ‘EzColocalization’ (https://github.com/DrHanLim/EzColocalization) following their respective instructions, and intensity and Manders’ coefficient data were used for further analysis.

#### Single cell RNA-sequencing analysis of human kidney

Normal human kidney snRNA-seq datasets were obtained from CZ CELLxGENE Discover.^69^ Relevant datasets representing normal kidney tissue were selected across multiple studies and analyzed within the platform to retrieve *BICC1* expression matrices and accompanying cell-type annotations. Expression of *BICC1* was extracted at the annotated cell-type level. Cell types were grouped into broader biological categories. For each cell type, the proportion of cells expressing BICC1 was calculated, and a dot plot was generated to display the percentage of cells expressing the gene, with color indicating scaled expression levels and dot size representing the fraction of cells expressing the gene. Analysis and visualisation were performed in R.

## Supplemental information

Document S1. Figures S1–S12, Table S1, and supplementary references

Data S1. Proteins enriched by co-purification with HA-tagged BICC1 from HEK293 cells

Data S2. Whole transcriptome sequencing and GSEA of KOs vs. UCL93
