# Supplementary material for: An integrin beta-1-anchored bicaudal C1-polycystin-1 module essential for tubular morphogenesis in polycystic kidney disease
Source: iScience. 2026 Aug 17;29(9):117209. doi: 10.1016/j.isci.2026.117209 (PMC13508657; doi:10.1016/j.isci.2026.117209)
Supplement: Supplementary file 1 [file mmc1.pdf]

iScience, Volume *29*

Supplemental information

An integrin beta-1-anchored bicaudal C1-polycystin-1 module essential for tubular morphogenesis in polycystic kidney disease

Andrew J. Streets, Manoj K. Valluru, Devon Smith, Lisa Chang, Iddo Z. Ben-Dov, Stuart A. Wilson, Christopher P. Toseland, and Albert C.M. Ong

SUPPLEMENTARY MATERIAL

Supplementary methods

Materials

All chemicals were purchased from Sigma Chemical (Poole, Dorset, United Kingdom), unless otherwise stated.

Antibodies

For IPs and western blotting, Primary antibodies used in this study were rabbit BICC1 (Sigma-Aldrich Cat# HPA045212), mouse BICC1 mAb (Santa Cruz Cat# sc-514846), PC1 mAb (7e12) [1], rabbit PC1 (2b7) [2], goat PC2 (Santa Cruz Cat# sc-10376), mouse ACTB (Abcam Cat# ab6276), HA High Affinity (rat monoclonal clone 3F10, Roche), mouse GST (Santa Cruz Cat# sc-138), rat c-Myc (clone JAC6, Biorad) and rabbit GFP (Cat# ab6556, Abcam). All primary antibodies were used at 1:1000 unless otherwise stated. Secondary antibodies used in this study include goat anti-mouse IgG (Cat# 1030-05, Southern Biotech), goat anti-rabbit IgG (Cat# 4050-01, Southern Biotech), goat anti-rat IgG (Cat# 3050-01, Southern Biotech) and rabbit anti-goat IgG (Cat# P0449, Dako). All secondary antibodies were used at 1:10,000, unless otherwise stated in the results section.

For staining, primary antibodies used were specific to rat HA High Affinity (1:300), rabbit BICC1 (1:200), mouse ARL13B (1:250, 66739-1-Ig, Proteintech), mouse/rabbit Pericentrin (1:250, ab28144 and ab4448, Abcam), rabbit CTNNB1 (1:250, 8480P Cell Signaling Tech), rabbit VCL (1:500, 26520-1-AP, Proteintech), mouse/rabbit PXN (1:500, 10029-1-Ig, Proteintech and 610051, BD Biosciences), rat ITGB1 (1:100, 9EG7 553715, BD Biosciences), mouse TLN1 (1:500, 8D4 ab157808, Abcam), rabbit TNS1 (1:250, NBP1-84129, NovusBio) and mouse/rabbit FBLIM1 (1:250, SC-271417, Santa Cruz Biotechnology and 13349-1-AP, Proteintech). Secondary antibodies AF488 or AF594 for either goat anti rabbit or mouse or rat (IgG (H+L) Highly Cross-Adsorbed Secondary Antibody, Alexa Fluor™, Invitrogen). All secondary antibodies were used at 1:500. Conjugates used in this study are Texas Red™-X Phalloidin (1:1000, T7471, Invitrogen) and Wheat Germ Agglutinin (WGA)-AF488 (1:1000, W11261, Invitrogen).

Cell Culture

UCL93 kidney epithelial cells were immortalized from primary cultures of tubular cells isolated from normal human kidneys removed for clinical indications as previously described [3]. Cells were grown in Dulbecco's modified Eagle's medium-Ham's 12 (DMEM-F12, Invitrogen) supplemented with 1% L-glutamine (Invitrogen), 5% NuSerum (Becton Dickinson), and 1% antibiotic/antimycotic solution

(Invitrogen) at 33°C/5% CO_2. HEK-293T cells were cultured in Dulbecco's modified Eagle's medium-Ham's 12 (DMEM-F12, Invitrogen) supplemented with 1% l-glutamine (Invitrogen), 10% FCS, and 1% antibiotic/antimycotic solution (Invitrogen) at 37°C/5% CO_2.

Generation and Validation of FLAG/HA-Tagged BICC1 HEK293 Inducible Cell Lines

To generate stable HEK293 cell lines that inducibly express FLAG/HA-tagged BICC1 isoforms 1 and 2, the Flp-In T-REx system and Gateway recombination cloning technology were utilized [4,5]. In brief, human BICC1 coding sequences were PCR-amplified from human podocyte cell line cDNA and cloned into a pENTR4 Gateway entry vector containing attL sites (Invitrogen). Two distinct inserts corresponding to isoforms 1 (excluding exon 21) and 2 (including exon 21), were identified by Sanger sequencing as described in mice [6] and confirmed by targeted PCR in ADPKD patient urine cDNA (not shown).

pENTR4 BICC1 isoform 1 was recombined into pFRT/TO/FLAG/HA-DEST destination vectors using GATEWAY LR recombinase to allow for doxycycline-inducible expression of stably transfected FLAG/HA-tagged protein from the TO/CMV promoter. Flp-In T-REx HEK293 cells (Invitrogen) were transfected with the expression constructs and pOG44 encoding the Flp recombinase in a 1:9 ratio then subjected to selection with hygromycin and blasticidin (to maintain TetR expression) to generate stable cell lines. Doxycyline-inducible expression of BICC1-isoform 1 was confirmed by Western blotting with an anti-HA antibody (Covance Cat# MMS-101P) and anti-BICC1 mAb (2D9, Sigma-Aldrich Cat# MABE344).

CRISPR/CAS9 mutagenesis

UCL93 cells were transfected with pSpCas9(BB)-2A-Puro (PX459) V2.0 (pSpCas9n(BB)-2A-Puro (PX462) V2.0 (Addgene plasmid #62988, kind gift of Feng Zhang) containing a gDNA targeting the first exon of *PKD1* (5'-CACCGCGCCGGGCGCTGGGCCGCAG), the first exon of *PKD2* (5'-CACCGCGTGGAGCCGCGATAACCC) or the first exon of *BICC1* (5'-CACCGGGAGAGCCCGGCTACCTGG). Positive clones were selected for puromycin resistance followed by limiting dilution. Mutations were then validated by genomic DNA sequencing and western blotting using specific antibodies to PC1 (7e12), PC2 (1A11) and BICC1 (Sigma-Aldrich Cat# HPA045212). The generation of *PKD1* null cells has been previously reported [7].

Lentiviral Rescue of Bicc1

N-terminal HA epitope tagged Bicc1 (Isoform 1, Q99MQ1-1) was cloned into pcW57.1 (Addgene plasmid #41393, kind gift of David Root). Viral particles were generated from HEK-293T cells following co-transfection of pcW57.1-HA-Bicc1 and MISSION Lentiviral Packaging Mix (Sigma, UK). Following incubation, stably transduced UCL93 BICC1 knockout cells were selected for puromycin resistance followed by limiting dilution. Inducible expression of HA-Bicc1 in *BICC1* and *BICC1/PKD1* knockout cells was initiated following incubation with 1µg/ml doxycycline (Sigma, UK) for 24 or 48h. BICC1 point mutations (human reference) were generated in HA-Bicc1 by site-directed mutagenesis as previously described [8].

Attachment, detachment and spreading assays

For attachment and spreading assays, cells were passaged and 10000 cells/well allowed to attach for 30 min. Unattached cells were washed away with PBS and remaining cells counted and cell area calculated using ImageJ. For cell detachment assay cells were plated in 96 well plates at 1000 cells/well for 48h. Cells were incubated with 1mM EDTA/PBS for 30 min and attached cells stained with 0.02% crystal violet for 30 min. Excess dye was discarded by washing the plates for 15 min in fresh water. The cell-bound dye was redissolved in 70% (v/v) ethanol/water and the optical density was measured at λ = 570 nm.

Matrigel 3D cyst assays

3D Matrigel cyst assays were performed as previously described [9]. In brief, UCL93 cells ($1x10^5$/well) were mixed with 70 µl Matrigel (Becton Dickinson, UK), plated into 96 well plates in triplicate and incubated for 30 min at 37°C to facilitate gel formation. Cells were then cultured for 12d in the presence of vehicle (DMSO) or 50µM CK-666 (Tocris, UK). Media was replaced every 2 days. The percentage of tubular structures was calculated from 3 independent wells per treatment.

Transfections

Cells were transfected using Lipofectamine 3000 (Life Technologies) or Amaxa electroporation (program W-01) for 48 hours before the cell assays. For siRNA knockdown assays, cells were transfected with negative control or specific ITGB1 siRNAs (SmartPool, Dharmacon, USA) using RNAimax (Life Technologies). siRNA knockdown was confirmed by qPCR using specific Taqman probes. mEmerald-Migfilin-C-14 was a kind gift from Michael Davidson (Addgene plasmid # 54181).

RT-qPCR

Relative mRNA expression was determined by SYBR green qPCR according to the manufacturer's protocol (Life Technologies). Following RNA extraction, cDNA was synthesised using a total RNA to cDNA kit (Life Technologies). Real time PCR was carried out on a QuantStudio 7 qPCR machine. Normalisation of expression was carried out using specific primers to actin. Relative quantification carried out using the comparative Ct (ΔΔCt) method to calculate fold changes in expression between samples.

Protein Biochemistry

Cells were lysed by extraction at 4°C using the IP lysis Buffer (25 mM NaCl, 150 mM EDTA, 1 mM 0.5% NP40, 1% Triton X-100, pH 7.0) supplemented with a protease inhibitor cocktail (Roche). Immunoblotting and immunoprecipitation were performed as previously described [2]. To identify proteins bound to actin pulldown was carried out using biotin-XX phallodin (Thermo-Fisher Scientific, UK) as described [10]. Briefly cell lysates were incubated overnight with 2mM biotin-XX phalloidin followed by capture with avidin Dynabeads C1 for 30 min. Lysates were washed with IP lysis buffer, 500mM NaCl and bound proteins detected by Western blot.

Due to concerns regarding antibody specificity and sensitivity, we employed membrane fractionation to confirm both plasma membrane and cytoplasmic components of endogenous BICC1. A plasma membrane protein extraction kit (Abcam, UK ab65400) was used to isolate plasma membrane and cytosolic protein fractions according to the manufacturer's instructions. For protein degradation assays, cells were incubated with 10µM Mg132 or 100nM BafA1 for 16h. Bio-Rad ChemiDoc XRS+ and Image Lab 5.1 software were used for visualization and quantification of proteins of interest. All quantification was carried out on non-saturated bands as determined by the software from 3 independent experiments.

LC-MS/MS analysis

During the sample preparation phase, HEK293 T-Rex (Control) and HEK-293 T-Rex cells stably expressing HA-BICC1 (Isoform 1, Q9H694-1) were plated at $1x10^5$ cells/mL and grown to 90% confluency. BICC1 and its protein binding partners were immunoprecipitated from cell lysates using anti-HA antibody beads. To test the efficiency of the Co-IP, a Western blot experiment was performed during the standardisation phase. LC-MS was done at the Fingerprints Proteomics department at the University of Dundee. Peptides were extracted from 1D SDS-PAGE gel slices and subjected to tryptic digestion. The trypsinised digests of each gel section were run on a 1D NanoLC ESI MS/MS instrument. The sample peptides are separated within the Capillary LC system, using a reverse phase C18 column.

The peptides are then eluted directly into the MS/MS instrument and detected as ions in the MS mode or further fragmented and detected in MS/MS mode. Samples were analysed using Mascot (Matrix Science, Boston, MA, USA), protein scores from HA-Bicc1 (n=3) and control (n=3) were merged and missing values were imputed by fitting a minimal value to a normal distribution. Further merged data was quantile normalised for differential enrichment analysis using R package DEGandMore (NormQQ and StudentsT Method, https://github.com/zhezhangsh/DEGandMore), the p-value of <0.05 being considered significant. Significantly enriched candidates were then classified as high-confidence interacting proteins (HCIPs) or non-specific binders in combination with the CRAPome contaminant database [11].

Total RNA sequencing (RNA-Seq) and analysis

UCL93 and KO cells were plated at $5x10^5$ cells/mL in 10 cm^2 plates and incubated at 33°C until 70% confluency was reached. Total RNA was extracted from the cells (n=3/ cell line) using a Trizol reagent. After rRNA removal, the NGS libraries were constructed and sequenced (Paired-end 150 bp) at the Novogene facility. Data was processed in ShARC (Sheffield Advanced Research Computer at the University of Sheffield). Quality control checks on raw sequence data were done using the bioconda package fastqc-0.11.8-1. The adapter trimming was performed using cutadapt-1.18, and reads were aligned to the human genome (human genome GRCh38 and annotations gencode.v29) using star=2.6.1 and Transcriptome.out.bam was realigned and quantified using rsem=1.2.28. Expected counts and TPM values were extracted from gene-level expression estimates files for further analysis. The expected counts were subjected to DESeq2 normalisation and two-group differential expression analysis using the R package DEGandMore, the p-value of <0.05 being considered significant. Gene Set Enrichment Analysis (GSEA) was conducted using the toolkit WebGestaltR, by selecting functional databases Geneontology, KEGG and Reactome.

Immunofluorescence and imaging

Semi-confluent UCL93 and KO cells on coverslips or plates were fixed with 4% paraformaldehyde or 10% Formalin in DPBS for 10 minutes. Cells were then washed and permeabilized in 0.5% Triton X-100 in DPBS with calcium and magnesium for 5-15 minutes. Cells were blocked with 3% BSA for 1 hour, incubated for either 1 hour at room temperature or overnight at 4°C with primary antibodies, and incubated for 1 hour with secondary antibodies. After washes, coverslips were mounted using ProLong™ with NucBlue™ Stain and imaged on a ZEISS Celldiscoverer7 Widefield Fluorescence Microscope, ZEISS LSM 980 with Airyscan 2 and Olympus Inverted IX71 fluorescence microscope (Light Microscope Facility, The University of Sheffield). For Airyscan imaging, 405, 488 and 561 nm laser lines

were used and fixed cells were imaged with a Plan-Apochromat 63×/1.4 numerical aperture (NA) oil objective on a ZEISS LSM 980 with Airyscan 2. For all fixed-cell imaging, images shown are snap images and maximum intensity z projections, unless otherwise noted. Images were acquired using ZEISS ZEN Microscopy Software and SimplePCI imaging software (Compix, Hamamatsu, PA). Airyscan images were further processed in ZEISS ZEN 3.0 blue edition software.

Actin directionality, proportional analysis and thickness

To investigate changes in directional orientation of actin stress fibres, Fourier component analysis for directionality (Goodness of Gaussian fit and angular dispersion of the actin stress fibres) was performed on F-actin channel ROI single cell images using the ImageJ plug-in 'Directionality' created by Jean-Yves Tinevez (https://imagej.net/plugins/directionality) following their respective instructions. Numerical values of the stress fibre direction, angular dispersion and goodness were exported to an Excel spreadsheet for further analysis. The main actin phenotype was quantified as the proportion of cells with disorganised stress fibres (DSFs) with higher angular dispersion and poor gaussian fit or the presence of actin asters. Pathtrace view of actin organisation was generated using AGAVE v1.1.0 (https://github.com/allen-cell-animated/agave). The thickness of the actin cortex, measured in micrometers (μm), was determined from images of Phalloidin-stained cells. This measurement was performed manually by drawing a line across the actin cortex using the line profile tool in Zen Blue 3.0.

Cilia quantification and proportion analysis

For cilia length measurements, ImageJ analysis software (NIH) was used to measure >100 cilia in at least 3 independent experiments. For proportion analysis, images of cells were collected using 10x magnification on a ZEISS Celldiscoverer7 Widefield Fluorescence Microscope and captured images were pre-processed for noise reduction and cilia segmentation. Next, particle Analysis was employed to identify individual cilia based on their size and shape (circularity <0.5). Converted masks were used to quantify the number of cilia in ROIs and % cells with single cilium. To investigate changes in directional orientation of cilia, Fourier component analysis for directionality was performed on ARL13B channel ROI images using the ImageJ plug-in 'Directionality' following their respective instructions. Numerical values of the cilia main direction, angular dispersion and goodness were exported to an Excel spreadsheet for further analysis.

Atomic Force Microscopy (AFM)

AFM measurements were performed with a Bruker Bioscope Resolve mounted on an inverted microscope (Nikon Eclipse Ti2) connected to an ORCA-Flash4.0LT (Hamamatsu) camera. Cells were seeded on 50 mm cell culture dishes (Willco GWST-5040) and allowed to adhere at 20% confluency for imaging. Imaging was performed in filtered PBS at room temperature. Pre-calibrated Silicon Tip – Nitride cantilevers (PFQNM-LC-V2 Bruker) were used with a 70 nm tip radius. PeakForce QNM/PeakForce Tapping with a maximum indentation force of 0.5 nN was used. Indentation curves were fitted within Nanoscope Analysis (Bruker) using a cone-sphere model [12 13] and an R^2 selection of >0.7. 10 regions of interest were selected for each cell which represents a total of 10,000 force curves per cell to calculate the Young's Modulus.

Traction force microscopy

Traction force microscopy (TFM) experiments were performed as previously described [14]. Traction force microscopy experiments were performed using collagen-coated 1 kPa hydrogels with 0.2 µm red fluorospheres Matrigen SoftTrac™ 35mm dishes (Cellguidance-Cambridge). Dishes were equilibrated with cell culture medium for 30 min at 33°C. UCL93 and KO cells were seeded and allowed to adhere overnight at 20% confluence for imaging. Phase images of attached cells and fluorescent embedded beads were collected using 40x magnification on a ZEISS Celldiscoverer7 Widefield Fluorescence Microscope. Images of the fluorescent microspheres on a relaxed hydrogel were collected after 5 min treatment with a 0.5% (w/v) Triton-X-100, 20 mM NH_4OH PBS solution to remove cells. The displacement of beads before and after the removal of cells was tracked by particle imaging velocimetry followed by Fourier transform traction cytometry to estimate the corresponding cell traction force field [15,16]. The total traction forces (in N) were measured by integrating traction forces over the cell area. The elastic energy (in J) stored in the gel to produce the observed deformation was calculated by summing the products of displacement with the force over the cell area.

Single cell migration assay

UCL93, and KO cells were seeded and allowed to adhere at 20% confluence (6 hours), cells were incubated with NucBlue™ Live ReadyProbes™ Reagent (Hoechst 33342) (added to cell culture), after 1-hour cells were washed with PBS and replaced with fresh media for Time-lapse imaging. Phase contrast and Hoechst 33342 images of attached were collected using 20x magnification on a Zeiss Celldiscoverer7 Widefield Fluorescence Microscope for every 12 minutes up to 12 hours (Carl Zeiss Microscopy, Thornwood, NY). Captured images were pre-processed for noise reduction and contrast enhancement. Cell tracking was performed using Fiji/ImageJ software with the TrackMate plugin created by Jean-Yves Tinevez (https://imagej.net/plugins/trackmate/) following their respective

instructions. Cell nuclei or phase contrast cell image were identified using segmenter called trackmate-thresholding-detector and TrackMate-clij2. The TrackMate algorithm called Kalman tracker was then employed to track individual cells over time based on their centroids. Trajectories of individual cells were analysed using TrackMate Analysers. Using optimised parameter file, all the images from three independent replicates were batch processed using the TrackMate Batcher. Trackmate files were merged using inbuilt merge function and exported for MotilityLab/celltrackR directionality analysis (https://github.com/ingewortel/celltrackR). Track data from multiple files was merged for further statistical analysis. Track displacement: Measure the distance between the last spot of the track and the first spot of the track in time. Confinement ratio: how "efficient" was a track displacement in getting far away from its starting point, it is defined as the net-displacement divided by the total-distance, values close to 0 indicate a confined movement, where the particle would stay close to its starting point and values close to 1 indicate that the particle travels along a line with a constant orientation. The net-displacement is given by the Track displacement feature, and the total distance is given by the Total distance travelled feature.

Low Formaldehyde RNA Immunoprecipitation

Formaldehyde RNA immunoprecipitation (fRIP) was carried out as previously described [17]. Protein G Dynabeads (Novex by Life Technologies) were used to immunoprecipitate endogenous BICC1 with 4 µg of anti-mouse BICC1 mAb (Santa Cruz Biotechnology Inc) or non-immune mIgG. The specificity of the BICC1 antibody for endogenous BICC1 is shown in Fig S2D. The beads/antibody suspension was incubated at room temperature for 1 hour before being washed twice with 900 µL lysis buffer. The remaining volume of the cleared lysate (approximately 300 µL) was added to the beads and the sample were rotated at 4°C for 2 hours. The samples were washed twice with 900 µL lysis buffer followed by washes with 900 µL high salt lysis buffer (0.5 M NaCl) and then washes with 900 µL normal lysis buffer. Following these washes, 56 µL ice-cold RNase free H_2O was added to the beads and 26 µL to the inputs, before 33 µL of the 3X reverse-crosslinking buffer (3X PBS, 6% N-lauroylsarcosine, 30 mM EDTA, 15 mM DTT), 10 µL proteinase K (Roche 19 mg/uL) and 1 µL RNase inhibitor was added. The beads were resuspended by flicking the tube gently and were then incubated at 42°C for 1 hour followed by 55°C for 1 hour with shaking at 1100rpm. The samples were placed on a magnet and the eluates were collected and the total volume was made to 250 µL (addition of approximately 150 µL) with ice-cold RNase free H2O. RNA was isolated and treated with DNase I. RT-qPCR was carried out using specific primers to candidate genes: *PKD1, PKD2, BICC1, FBLIM1, TNS1, ACTB*.

Proximity Ligation Assay (PLA) and NSPs plus PLA (Puro-PLA)

For In situ detection of endogenous BICC1 and ITGB1 interactions by PLA, UCL93 and KO cells cultured semi-confluent on coverslips fixed and incubated with mouse anti-BICC1 (1:250) and rabbit anti-ITGB1 (1:250) antibody overnight. For Puro-PLA, cells were incubated with puromycin (10µg/ml, up to 12 min) for the condition and with cycloheximide (100µg/ml, up to 3 hours) plus puromycin (10µg/ml, up to 12 min) for the controls, treated cells were fixed and incubated with mouse anti-puromycin (1:500) and rabbit anti-FBLIM1 (1:250) antibodies overnight. Cycloheximide (CHX) is a potent inhibitor of ribosome-mediated translation elongation. It halts the ribosome before puromycin can be incorporated into nascent polypeptide chains, serving as a negative control [18]. PLA was performed using Duolink Reagents (DUO92008, Merck) following the manufacturer's instructions [19]. Secondary antibodies Duolink® In Situ PLA® Probe Anti-Rabbit PLUS and Anti-Mouse MINUS (DUO92002 and DUO92004, Merck) were used at 1:5. Cells were incubated with Wheat Germ Agglutinin (WGA)-AF488 (1:1000) for 10 min before the last wash and mounted using ProLong™ with NucBlue™ Stain. Images of cells were collected using 20x and 63x magnification as apical-basal z-stack or focused to the basal side of cells on a ZEISS LSM 980 with Airyscan 2.

Data visualisation and statistical analysis

Data are presented as mean values ± SEM. Parametric Student's t-test and one-way ANOVA were used for statistical analysis with a p-value of <0.05 indicating statistical significance (*p ≤ 0.05, **p ≤ 0.01, ***p ≤ 0.001 and ****p ≤ 0.0001). Plotting and analyses were carried out using Prism 9 (Graphpad). Heat maps were generated using morpheus.R (https://github.com/cmap/morpheus.R) and Volcano plots were created using VolcaNoseR (https://github.com/JoachimGoedhart/VolcaNoseR).

Cytoscape network analysis

Protein-protein interaction and gene network analysis was conducted for proteins or genes of interest using a StringApp in Cytoscape v3.9. Network Nodes were colour-coded for differential expression (RNA-seq) or enrichment (MS), edges were colour-coded by co-expression scores and thickness by confident scores from the STRING v12 database. The network was exported to Draw.io and overlaid on a representative cell adhesion image generated using Bioicons seriver CC BY 4.0 (https://github.com/duerrsimon/bioicons).

Actin and adhesion proteins (AAPs) data curation and analysis

We curated a list of AAPs from several published datasets (n=496, Fig S6). Three major datasets were an F-actin interactome obtained by phalloidin pull-down [20], a *b-Actin* mRNA interactome and integrin adhesion complexes (IACs) by proximity biotinylation and mass spectrometry [21,22]. We compared these

AAPs with differentially expressed genes detected in the KOs by analysing the relationships between sets and their intersections. UpSet plots were created using intervene (https://github.com/asntech/intervene). A Kidney, Liver and Brain enhanced RNA interactome capture (eRIC) dataset was analysed to identify potential associations with BICC1 interacting IACs identified in this study [23].

Segmentation and colocalization analysis

Image analysis was conducted using ImageJ/Fiji (https://imagej.net/) and Napari (https://napari.org/). Airyscan-processed focal adhesion images were pre-processed to enhance contrast and reduce noise using background subtraction and Gaussian blurring. To segment focal adhesions, the default dark thresholding method was applied to binarize the image, followed by morphological operations such as erosion and dilation to remove noise and refine object boundaries. Next, particle analysis was employed to identify individual focal adhesions based on their size and shape. Additional features such as circularity (<0.5) and aspect ratio were used to discriminate against non-specific structures. Converted masks were used for further analysis such as to quantify number of FA sites including the intensity of protein expression in segmented FA sites. Nuclear objects were segmented using the ImageJ plug-in 'StarDist' (versatile fluorescent nuclei, https://imagej.net/plugins/stardist) following their respective instructions. Cells were segmented using the ImageJ plug-in 'Cellpose using the BIOP-EPFL' (https://github.com/BioImaging-NKI/Cellpose-Fiji) or napari plug-in 'serialcellpose' (https://www.napari-hub.org/plugins/napari-serialcellpose) following their respective instructions. Colocalization analysis was conducted using the ImageJ plug-in 'EzColocalization' (https://github.com/DrHanLim/EzColocalization) following their respective instructions, and intensity and Manders' coefficient data were used for further analysis.

Tissue Immunofluorescence

Frozen tissue sections were placed in 4% paraformaldehyde in PBS for 20 min and sections were washed with PBS twice for 5 min. The tissue sections were then permeabilised with 0.1% v/v Tween-20 (Sigma-Aldrich, St. Louis, MO, USA) in PBS for 10 min and washed again with PBS for 5 min. Paraffin embedded kidney sections were dewaxed and rehydrated before antigen unmasking in a boiling citrate solution (1 mmol/l Tri-sodium citrate, pH 6.0, VWR) for 10 minutes All tissue sections were then blocked with 2% w/v BSA in PBS (incubation buffer) for 20 min. For actin filament staining, the frozen sections were incubated with a 1:20 dilution of phalloidin in incubation buffer for 60 min. After the incubation, the sections were washed three times with PBS, 5 min each. For FA markers paraffin

embedded sections were incubated overnight with primary antibodies to FBLIM1 (Santa Cruz) or ITGB1 (Proteintech). After 3x5min PBS washes sections were incubated with Alexa Fluor 488 secondary antibodies (Invitrogen, USA) for 1h. After washes coverslips were mounted using ProLong™ with NucBlue™ Stain and imaged on ZEISS LSM 980 with Airyscan 2 (Light Microscope Facility, The University of Sheffield). Airyscan images were further processed in ZEISS ZEN 3.0 blue edition software.

In vivo experiments

All experiments in mice were carried out under project licence PP9820851 with local ethical approval. *Pkd1* deletion was induced by doxycycline injections at postnatal days (PNs) 13-15 in tetracycline-inducible, kidney-specific *Pkd1* mice (*Pax8rtTA-TetO-Cre-Pkd1fl/fl*). After sacrifice at PN23, *Pkd1* ko and WT control kidneys were collected and embedded in cry-M-bed solution (Wolflabs) or immersed in 10% neutral buffered formalin (Millipore) for histological analysis. Tissue sections were processed for immunofluorescence staining. *Bicc1*$^{-/-}$ kidney tissue sections (E18.5) were the kind gift of Prof Daniel Constam (École Polytechnique Fédérale de Lausanne, Switzerland).

Single cell RNA-sequencing analysis of human kidney

Normal human kidney snRNA-seq datasets were obtained from CZ CELLxGENE Discover [24]. Relevant datasets representing normal kidney tissue were selected across multiple studies and analysed within the platform to retrieve *BICC1* expression matrices and accompanying cell-type annotations. Expression of *BICC1* was extracted at the annotated cell-type level. Cell types were grouped into broader biological categories. For each cell type, the proportion of cells expressing BICC1 was calculated, and a dot plot was generated to display the percentage of cells expressing the gene, with colour indicating scaled expression levels and dot size representing the fraction of cells expressing the gene. Analysis and visualisation were performed in R. Normal human kidney snRNA-seq reference gene sets were obtained from Lake et. al. [25]. Gene expression profiles from UCL93 cells (n = 3; TPM values) selected by reference cell-type signature and visualised as a heat map. A dot plot was generated to illustrate the expression of selected nephron segment-specific marker genes across the UCL93 samples.

Supplementary Figures

Figure S1: Single cell RNA-sequencing of *BICC1* expression in human kidney cell types

(A) Dot plot showing the percentage of cells expressing *BICC1*, colour-coded by scaled expression level and grouped by broad cell categories. Little to no expression was observed in blood cells or the stromal/endothelial fraction. In the glomerulus, high expression was restricted to parietal epithelial cells. Within the nephron, strong expression was observed in the proximal tubule, the descending limb of the loop of Henle, the distal convoluted tubule, and the connecting tubule. PT - proximal tubule, PCT - proximal convoluted tubule, PST - proximal straight tubule, LOH AL/DL – ascending or descending limb of the loop of Henle, DCT- distal convoluted tubule, CNT - connecting tubule, CD – collecting duct. Normal human kidney snRNA-sequencing datasets were obtained from CZ CELLxGENE Discover [24].

Figure S2. UCL93 cells exhibit a proximal tubule-like gene expression signature.

Heat map showing gene expression (Z-score of TPM) from UCL93 samples (n = 3), annotated using a human kidney single-cell reference gene set [25]. UCL93 cells exhibit the highest enrichment for the PT-5 proximal tubule cluster, corresponding to the S3 segment, with >90% of PT-5 segment-specific genes being highly expressed. A small overlap (<30%) with PT-4 and DTL gene clusters was detected.

Dot plots show the mean expression ± SD of proximal tubule markers (ALDH2, SLC5A2, ANPEP, and AQP1), the collecting duct marker (AQP2), and the loop of Henle marker (UMOD). UCL93 cells display robust expression of proximal tubule markers, whereas AQP2 and UMOD expression is absent or negligible.

Figure S3: **Reciprocal stabilisation of BICC1 and PC1, specificity of BICC1 antibody, disruption of F-actin polymerisation and cyst formation.**

(A) Quantitation of western blot analysis for PC1 or BICC1 in selected *BICC1* and *PKD1* KO clones (n=5). Protein expression was normalised to calnexin.

(B) Immunofluorescence staining of UCL93 and *BICC1* KO cells with a rabbit BICC1 antibody (Sigma-Aldrich Cat# HPA045212) demonstrating specificity for endogenous BICC1 (red). The arrow indicates plasma membrane localised BICC1 in a single cell.

(C) Inducible expression of HA-Bicc1 in stable lentiviral *BICC1* null cells following induction with 1µg/ml doxycycline for 24h. Blots were probed with β-tubulin as a loading control (left panel). Immunofluorescence staining with an HA antibody before or after induction with doxycycline (right panel).

(D) UCL93 cells typically form tubular branching structures in 3D Matrigel. The Arp2/3 inhibitor CK666 known to inhibit F-actin branching induced cyst formation under the same conditions. Representative images of tubular structures and cysts after 12 days treatment. The percentage of tubular structures was reduced at 50 µM (n = 3 independent experiments, N = 20 individual fields per treatment condition) and 100 µM (now shown). One way ANOVA was performed to assess statistical significance (p value **** <0.0001).

Figure S4: Stress fibre organisation in confluent UCL93, PC1KO and BICC1KO cells.
Representative images of confluent UCL93, PC1KO and BICC1KO cells stained with Phalloidin (red or grey LUT) to visualise F-actin and Hoechst 33342 (blue) to label nuclei. Higher-magnification images highlight stress fibre organisation in each cell line. UCL93 cells exhibit well-organised actin stress fibres, whereas PC1KO and BICC1KO cells display disrupted and disorganised stress fibre architecture (arrows).

Figure S5. Subcellular localisation of BICC1 or HA-Bicc1 to cytoplasmic granules, paxillin and ITGB1-positive focal adhesion sites.
(A) Representative immunofluorescence images of UCL93 and BICC1KO cells stained for BICC1 (green) and nuclei (Hoechst 33342, blue). As expected, no BICC1 signal was detected in BICC1KO cells. Higher-magnification images show the distribution of BICC1 under basal conditions present as small cytoplasmic granules.
(B) Representative immunofluorescence image of UCL93 cells stained for BICC1 (green) and Paxillin (magenta). Higher-magnification images demonstrate the colocalization of BICC1 with Paxillin-positive focal adhesion sites (arrows).
(C) Representative immunofluorescence image of HA-Bicc1 (green) and Integrin β1 (ITGB1; orange) in BICC1 KO cells stably expressing HA-Bicc1. Higher-magnification image and the corresponding line profile plot (right) demonstrate the colocalization of HA-Bicc1 with ITGB1 at the leading edge of the cell.

Figure S6: BICC1 and F-actin form a complex in kidney epithelial cells.
(A) Biotinylated-Phalloidin pulldown of endogenous BICC1 in UCL93 cells using avidin beads confirming their direct interaction. BICC1 KO cells were included as a negative control. Data is presented as a representative blot of 3 independent experiments.
(B) Diagram of myc-Bicc1 and selected truncation Bicc1 mutants tested by biotinylated-Phalloidin pulldown in transfected HEK-293 cells using avidin beads.

(C) Biotinylated-Phalloidin pulldown experiments showed loss of interaction solely in the KH BICC1 mutant but preservation in the SAM deletion and partial IVS deletion mutants (del 133-691) which has an intact N-terminal sequence which contains the KH1 domain (aa1-132), the C-terminal SAM domain and part of the IVS (aa692-977). Data shown is a representative blot of 3 independent experiments.

Figure S7: **Endogenous BICC1 and HA-Bicc1 co-localised with the centrosomal protein pericentrin but are absent from primary cilia (ARL13B).**

(A) Representative images of endogenous BICC1 and ARL13B labelling in UCL93 and *BICC1*KO cells. BICC1 was absent from primary cilia (ARL13B) in both the z-stack maximum projection and individual stack images. *BICC1*KO cells (clone c4) served as a negative control.

(B) HA-Bicc1 (left panel) and endogenous BICC1 (middle panel) localised to both centrosomes labelled with pericentrin. Co-localisation was detected in both the z-stack maximum projection and individual stack images. There was no BICC1 signal in *BICC1*KO cells (right panel).

(C) Representative images of β-catenin (cell junctions) and ARL13B (cilia) labelled cells for UCL93 and *BICC1*KO, with segmented cilia from a cilia high content screen. There were no significant differences in the mean percentage of cells with single cilia between UCL93 and *BICC1*KO cells.

(D) A change in the cilia shaft orientation angle to 40° was observed in *BICC1KO* cells compared to UCL93 cells. Each data point is representative of a captured ROI with approximately >500 cells per ROI (UCL93= 99 ROIs and *BICC1*KO=92 ROIs).

(E) An increase in cilia length was observed in two clones (c3, c40) but no difference observed for four other clones (c4, c20, c35, c36) relative to UCL93 cells (>105 cilia per line). Data are presented as mean values ± SEM. One way ANOVA was performed to assess statistical significance (p values * <0.05, ** <0.01, *** <0.001, **** <0.0001 and ns = not significant).

Figure S8: **Changes in cell stiffness in *BICC1* and *PKD1* null cells measured by AFM.**

Cells were imaged using Atomic Force Microscopy (AFM) with the Peak Force Tapping mode. The image presents a typical UCL93, *PKD1* and *BICC1* knockout (KO) cells under a maximum indentation force of 0.5 nN. The images depict the Stiffness (Young's modulus E) and Height (μm) of the cells. Each selected point represents an average value for a region of interest (ROI) of the cells, calculated from 100-300 independent force curves, with over 10,000 force curves analyzed per cell line (R^2 > 0.7). Bar dot plots illustrate a significant increase in stiffness for *PKD1* KO cells compared to UCL93 cells, while *BICC1* KO cells show a trend towards increased stiffness (n=120-140 ROIs from n=13-14 cells per cell line).

Figure S9: **Curation of an Actin Associated Proteins (AAPs) Gene Set.**
A representative image of curated AAPs dataset from different published sources (n=431). Key references are indicated. The upset plot below indicates significant common differentially expressed AAPs between *BICC1*KO vs. UCL93 and *PKD1*KO vs. UCL93, highlighting 189 common differentially expressed AAPs in *PKD1* and *BICC1* KO cells.

Figure S10: **Protein expression of candidate FA proteins in *BICC1* and *PKD1* KO clones and the subcellular localisation of FBLIM1 in wild-type (UCL93) and KO clones.**
(A, B) Expression of focal adhesion proteins (FBLIM1, TNS1, vinculin) in three different *PKD1* KO clones (c1, c7, c17) or *BICC1* KO clones (c4, c20, c35). Blots were probed with actin as a loading control. Data presented is a representative blot of 3 separate repeat experiments.
(C) Z-stack images (Top panel) of FBLIM1 localisation at FA sites (PXN labelled) and cell-cell adhesion sites in UCL93 cells with reduced expression in *PKD1*KO clone (C1) and *BICC1* KO clone (C4). Widefield fluorescence microscope images (bottom panel) showing more clearly the reduction in intercellular staining in KO cells.

Figure S11: RNA-IP experiments detected key mRNAs bound to BICC1 in human kidney tubular cells and the visualisation of basolateral *FBLIM1* translation using a Puro-PLA assay.
(A) fRIP following pull-down with a BICC1 mAb in UCL93 cells. qPCR was used to detect selected mRNA transcripts (*BICC1, PKD1, PKD2*) co-purified with BICC1 in RIP experiments (n=4). *ACTB* served as a negative control. Fold enrichment was calculated relative to a negative fRIP control. Statistical significance by one way ANOVA (p values ** <0.01, **** <0.0001).
(B) Detection of mRNA for *FBLIM1, TNS1, BICC1* transcripts associated with BICC1 by qPCR in RNA-IP experiments (n=4). The RNA transcripts were normalised relative to control RIP experiments. *ACTB* mRNA served as a negative control. Data are presented as mean values ± SEM. One way ANOVA was performed to assess statistical significance (p values * <0.05, ** <0.01, **** <0.0001).
(C) qPCR showing increased expression of *FBLIM1* mRNA following induction of HA-Bicc1 expression with 1µg/ml doxycycline for 48 h (n=3).
(D) Diagram showing the principle of the Puro-PLA assay. YZ projections show newly synthesized FBLIM1 (red) in UCL93 cells clearly localised to the basolateral surface with a significant decrease in the number of PLA spots in Puromycin plus CHX treated controls.
(E) Representative wide-field images of cell cytoplasm labelled with WGA plus a Puro-PLA Signal demonstrating newly synthesised FBLIM1 (red) at the basal surface of UCL93, *PKD1*KO, and *BICC1*KO

cells. Very few PLA spots were observed in the KO cells compared to UCL93 cells. Cycloheximide (CHX) is a potent inhibitor of ribosome-mediated translation elongation and served as a negative control, Each data point is representative of a single cell (Basal PLA spots from Puro UCL93 n=192, *PKD1*KO n=132, *BICC1*KO n=183; Puro+CHX UCL93 n=240, *PKD1*KO n=183 and *BICC1*KO n=166 cells from 3 independent replicates). Data are presented as mean values ± SEM. One way ANOVA was performed to assess statistical significance (p values * <0.05, ** <0.01, *** <0.001, **** <0.0001 and ns = not significant).

Figure S12: F-actin organisation in PC2 KO cells and FBLIM1 expression in HEK-293T cells

(A) Representative images of UCL93 and PC2KO cells stained with Phalloidin (red) to visualise F-actin and Hoechst 33342 (blue) to label nuclei. Higher-magnification images highlight the organisation of stress fibres in both UCL93 and PC2KO cells.

(B) F-actin stress fibre organization in 3 representative BICC1 KO (C4, C20,C35), PKD1 KO (C1, C7, C17) and PKD2 KO (C20, C27, C38) clonal lines. The panel displays Phalloidin (red) stained cells, showing normal stress fibres in PKD2 KO cells; disorganised stress fibres (DSF) and actin asters (arrowheads) in BICC1 KO and PKD1 KO cells.

(C) Western blot showing relative expression of FBLIM1 in UCL93 and HEK-293T cells. The graph indicates that FBLIM1 expression in HEK-293T cells is 0.34 of that in UCL93 cells under standard culture conditions.

Supplementary Tables

Table S1: **Sequence analysis of *BICC1* and *PKD1* CRISP/Cas9 clones**

***PKD1* CrispR cell clone**	**DNA sequence change**	**Mutation effect**
UCL93 PC1 KO c1	c.53_150del	Frameshift from W^{18}
UCL93 PC1 KO c7	c.32_36del<br>c.104_117del	Frameshift from L^{11}<br>Frameshift from P^{35}
UCL93 PC1 KO c11	c.117_135del	Frameshift from C^{39}
UCL93 PC1 KO c17	c.106delG	Frameshift from C^{39}
UCL93 PC1 KO c23	c.29_204del	Frameshift from A^{10}
UCL93 PC1 KO c24	c.45_103del	Frameshift from L^{15}
**BICC1 CrispR cell clone**		
UCL93 BICC1 KO c3	c.26_27delAC	Frameshift from Y^{9}
UCL93 BICC1 KO c4	c.28insertA	Frameshift from L^{10}
UCL93 BICC1 KO c20	c.28delC	Frameshift from L^{10}
UCL93 BICC1 KO c35	c.29delT	Frameshift from L^{10}
UCL93 BICC1 KO c36	c.29delT	Frameshift from L^{10}
UCL93 BICC1 KO c40	c.29delT	Frameshift from L^{10}
**PKD2 CrispR cell clone**		
UCL93 PKD2 KO c15	c.386delC	Frameshift from S^{128}
UCL93 PKD2 KO c20	c.172_390 del	Frameshift from A^{62}
UCL93 PKD2 KO c33	c.365 delG	Frameshift from S^{122}
UCL93 PKD2 KO c37	c.358delA	Frameshift from N^{91}
UCL93 PKD2 KO c38	c.374delC	Frameshift from S^{125}

Supplementary references

1. Ong, A.C., Harris, P.C., Davies, D.R., Pritchard, L., Rossetti, S., Biddolph, S., Vaux, D.J., Migone, N., and Ward, C.J. (1999). Polycystin-1 expression in PKD1, early-onset PKD1, and TSC2/PKD1 cystic tissue. Kidney Int *56*, 1324-1333. 10.1046/j.1523-1755.1999.00659.x.
2. Newby, L.J., Streets, A.J., Zhao, Y., Harris, P.C., Ward, C.J., and Ong, A.C. (2002). Identification, characterization, and localization of a novel kidney polycystin-1-polycystin-2 complex. J Biol Chem *277*, 20763-20773.
3. Parker, E., Newby, L.J., Sharpe, C.C., Rossetti, S., Streets, A.J., Harris, P.C., O'Hare, M.J., and Ong, A.C. (2007). Hyperproliferation of PKD1 cystic cells is induced by insulin-like growth factor-1 activation of the Ras/Raf signalling system. Kidney Int *72*, 157-165.
4. Spitzer, J., Landthaler, M., and Tuschl, T. (2013). Rapid creation of stable mammalian cell lines for regulated expression of proteins using the Gateway(R) recombination cloning technology and Flp-In T-REx(R) lines. Methods in enzymology *529*, 99-124. 10.1016/B978-0-12-418687-3.00008-2.
5. Hafner, M., Landthaler, M., Burger, L., Khorshid, M., Hausser, J., Berninger, P., Rothballer, A., Ascano, M., Jr., Jungkamp, A.C., Munschauer, M., et al. (2010). Transcriptome-wide identification of RNA-binding protein and microRNA target sites by PAR-CLIP. Cell *141*, 129-141. 10.1016/j.cell.2010.03.009.
6. Cogswell, C., Price, S.J., Hou, X., Guay-Woodford, L.M., Flaherty, L., and Bryda, E.C. (2003). Positional cloning of jcpk/bpk locus of the mouse. Mamm Genome *14*, 242-249.
7. Streets, A.J., Prosseda, P.P., and Ong, A.C. (2020). Polycystin-1 regulates ARHGAP35-dependent centrosomal RhoA activation and ROCK signaling. JCI Insight *5*. 10.1172/jci.insight.135385.
8. Streets, A.J., Wessely, O., Peters, D.J., and Ong, A.C. (2013). Hyperphosphorylation of polycystin-2 at a critical residue in disease reveals an essential role for polycystin-1-regulated dephosphorylation. Human molecular genetics *22*, 1924-1939. 10.1093/hmg/ddt031.
9. Lannoy, M., Valluru, M.K., Chang, L., Abdela-Ali, F., Peters, D.J.M., Streets, A.J., and Ong, A.C.M. (2020). The positive effect of selective prostaglandin E2 receptor EP2 and EP4 blockade on cystogenesis in vitro is counteracted by increased kidney inflammation in vivo. Kidney Int *98*, 404-419. 10.1016/j.kint.2020.02.012.
10. Krippner, S., Winkelmeier, J., Knerr, J., Brandt, D.T., Virant, D., Schwan, C., Endesfelder, U., and Grosse, R. (2020). Postmitotic expansion of cell nuclei requires nuclear actin filament bundling by α-actinin 4. EMBO reports *21*. 10.15252/embr.202050758.
11. Mellacheruvu, D., Wright, Z., Couzens, A.L., Lambert, J.P., St-Denis, N.A., Li, T., Miteva, Y.V., Hauri, S., Sardiu, M.E., Low, T.Y., et al. (2013). The CRAPome: a contaminant repository for affinity purification-mass spectrometry data. Nat Methods *10*, 730-736. 10.1038/nmeth.2557.
12. Briscoe, B.J., Sebastian, K.S., and Adams, M.J. (1994). The effect of indenter geometry on the elastic response to indentation. J Phys D: Appl Phys *27* (*6*), 1156–1162. 10.1088/0022-3727/27/6/013.
13. Raquel González-Novo, Héctor Zamora-Carreras, Ana de Lope-Planelles, Horacio López-Menéndez, Pedro Roda-Navarro, Francisco Monroy, Lin Wang, Christopher P. Toseland, and Muñoz, J.R. (2026). 3D environment favours persistent changes in cell functions and altered morphology, wrinkling, and biomechanical signature of the

nucleus. Cell Reports Physical Science *7*, 103116. https://doi.org/10.1016/j.xcrp.2026.103116.

14. Lopez-Guajardo, A., Zafar, A., Al Hennawi, K., Rossi, V., Alrwaili, A., Medcalf, J.D., Dunning, M., Nordgren, N., Pettersson, T., Estabrook, I.D., et al. (2023). Regulation of cellular contractile force, shape and migration of fibroblasts by oncogenes and Histone deacetylase 6. Front Mol Biosci *10*, 1197814. 10.3389/fmolb.2023.1197814.
15. Martiel, J.L., Leal, A., Kurzawa, L., Balland, M., Wang, I., Vignaud, T., Tseng, Q., and Thery, M. (2015). Measurement of cell traction forces with ImageJ. Methods Cell Biol *125*, 269-287. 10.1016/bs.mcb.2014.10.008.
16. Lee, S., and Kumar, S. (2020). Cofilin is required for polarization of tension in stress fiber networks during migration. J Cell Sci *133*. 10.1242/jcs.243873.
17. G Hendrickson, D., Kelley, D.R., Tenen, D., Bernstein, B., and Rinn, J.L. (2016). Widespread RNA binding by chromatin-associated proteins. Genome Biol *17*. 10.1186/s13059-016-0878-3.
18. Schneider-Poetsch, T., Ju, J., Eyler, D.E., Dang, Y., Bhat, S., Merrick, W.C., Green, R., Shen, B., and Liu, J.O. (2010). Inhibition of eukaryotic translation elongation by cycloheximide and lactimidomycin. Nat Chem Biol *6*, 209-217. 10.1038/nchembio.304.
19. Soderberg, O., Gullberg, M., Jarvius, M., Ridderstrale, K., Leuchowius, K.J., Jarvius, J., Wester, K., Hydbring, P., Bahram, F., Larsson, L.G., and Landegren, U. (2006). Direct observation of individual endogenous protein complexes in situ by proximity ligation. Nat Methods *3*, 995-1000. 10.1038/nmeth947.
20. Serres, M.P., Samwer, M., Truong Quang, B.A., Lavoie, G., Perera, U., Gorlich, D., Charras, G., Petronczki, M., Roux, P.P., and Paluch, E.K. (2020). F-Actin Interactome Reveals Vimentin as a Key Regulator of Actin Organization and Cell Mechanics in Mitosis. Dev Cell *52*, 210-222 e217. 10.1016/j.devcel.2019.12.011.
21. Mukherjee, J., Hermesh, O., Eliscovich, C., Nalpas, N., Franz-Wachtel, M., Macek, B., and Jansen, R.P. (2019). beta-Actin mRNA interactome mapping by proximity biotinylation. Proc Natl Acad Sci U S A *116*, 12863-12872. 10.1073/pnas.1820737116.
22. Chastney, M.R., Lawless, C., Humphries, J.D., Warwood, S., Jones, M.C., Knight, D., Jorgensen, C., and Humphries, M.J. (2020). Topological features of integrin adhesion complexes revealed by multiplexed proximity biotinylation. J Cell Biol *219*. 10.1083/jcb.202003038.
23. Perez-Perri, J.I., Ferring-Appel, D., Huppertz, I., Schwarzl, T., Sahadevan, S., Stein, F., Rettel, M., Galy, B., and Hentze, M.W. (2023). The RNA-binding protein landscapes differ between mammalian organs and cultured cells. Nat Commun *14*, 2074. 10.1038/s41467-023-37494-w.
24. Program, C.Z.I.C.S., Abdulla, S., Aevermann, B., Assis, P., Badajoz, S., Bell, S.M., Bezzi, E., Cakir, B., Chaffer, J., Chambers, S., et al. (2025). CZ CELLxGENE Discover: a single-cell data platform for scalable exploration, analysis and modeling of aggregated data. Nucleic Acids Res *53*, D886-D900. 10.1093/nar/gkae1142.
25. Lake, B.B., Chen, S., Hoshi, M., Plongthongkum, N., Salamon, D., Knoten, A., Vijayan, A., Venkatesh, R., Kim, E.H., Gao, D., et al. (2019). A single-nucleus RNA-sequencing pipeline to decipher the molecular anatomy and pathophysiology of human kidneys. Nat Commun *10*, 2832. 10.1038/s41467-019-10861-2.

Figure S1

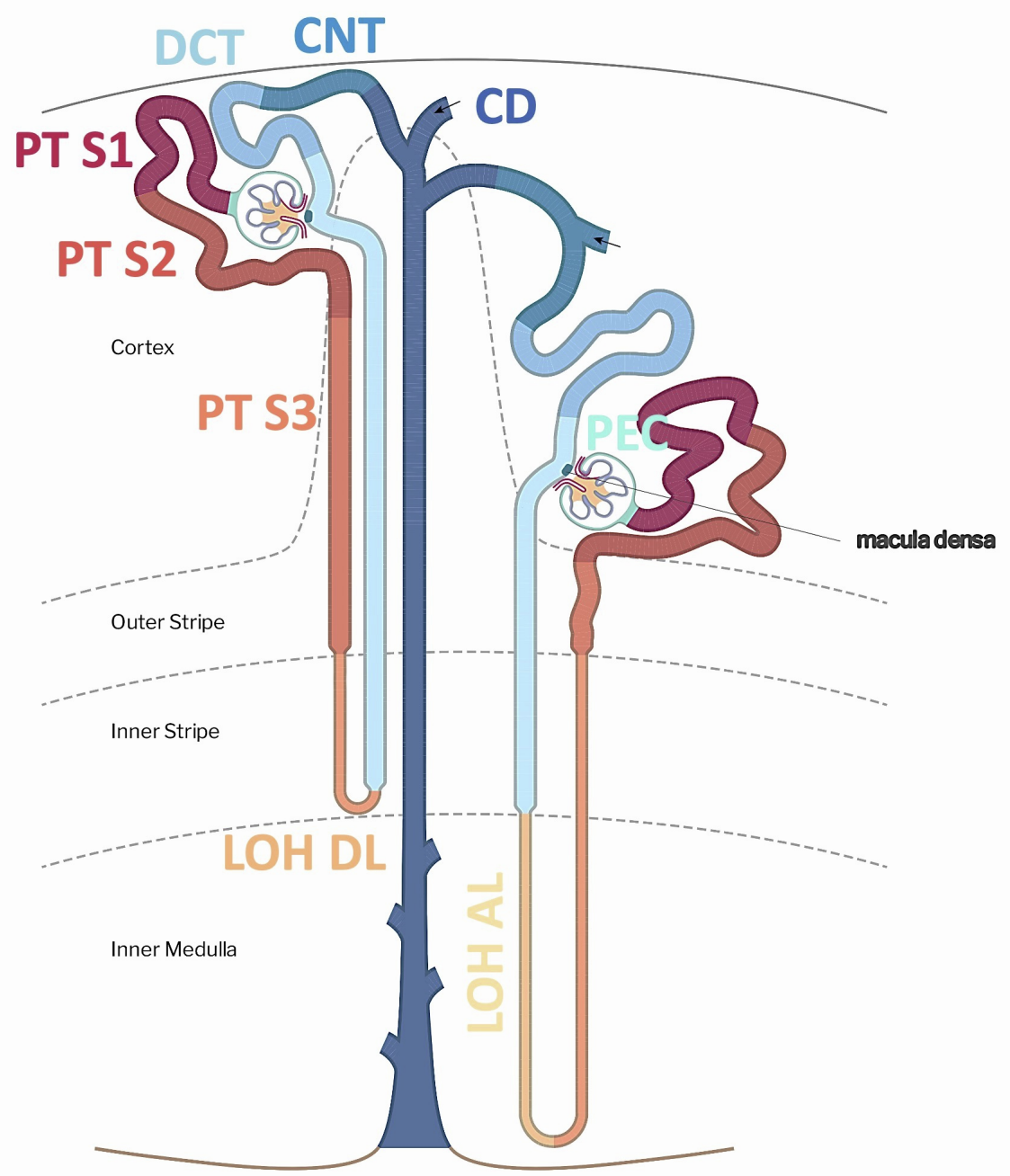

PEC – Parietal epithelial cells, **PT -** proximal tubule, **S** – Segment, **LOH AL/DL** – ascending limb of the loop of Henle, **LOH DL** – descending limb of the loop of Henle, **DCT** - distal convoluted tubule, **CNT** - connecting tubule, **CD** – collecting duct.

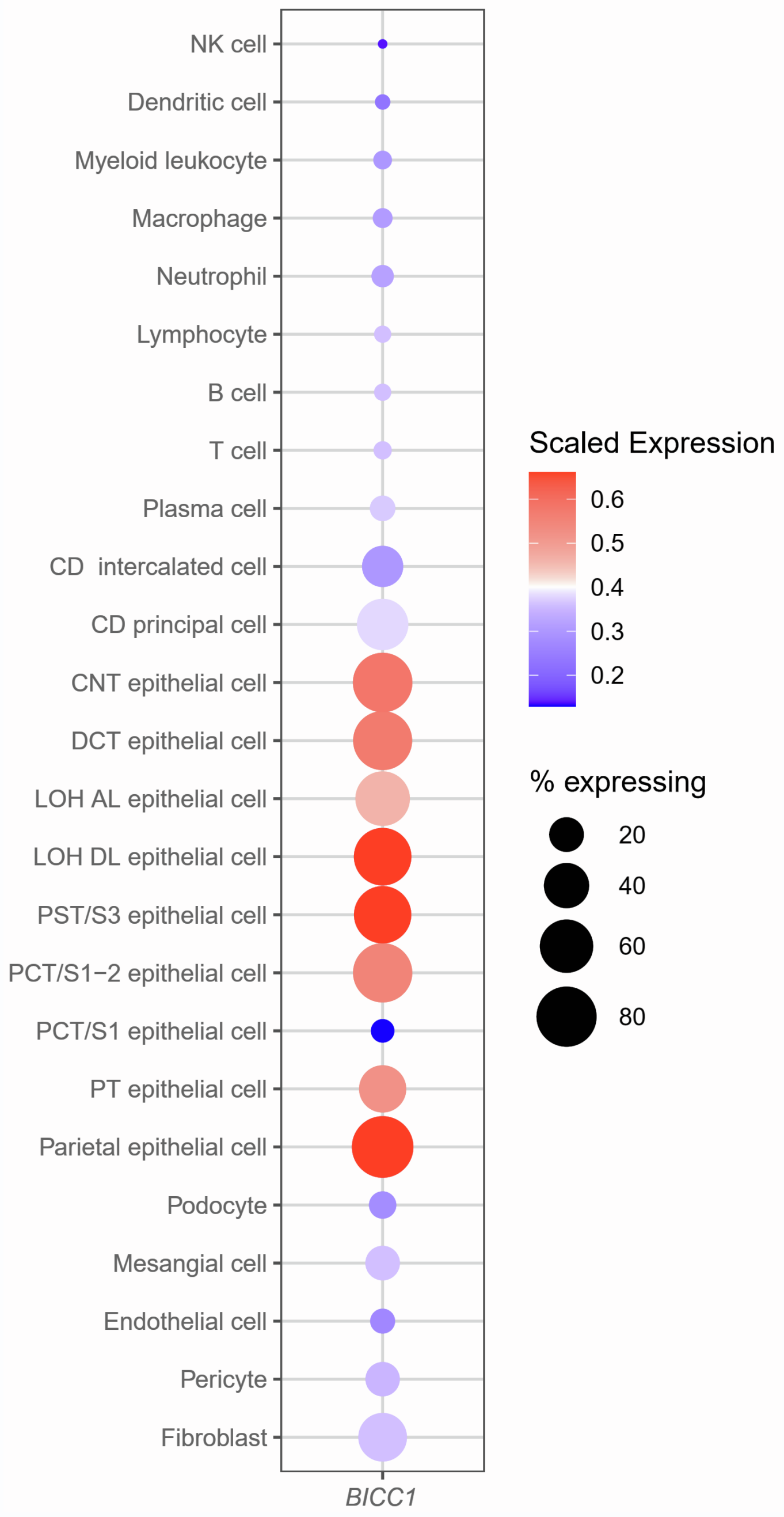

Figure S2

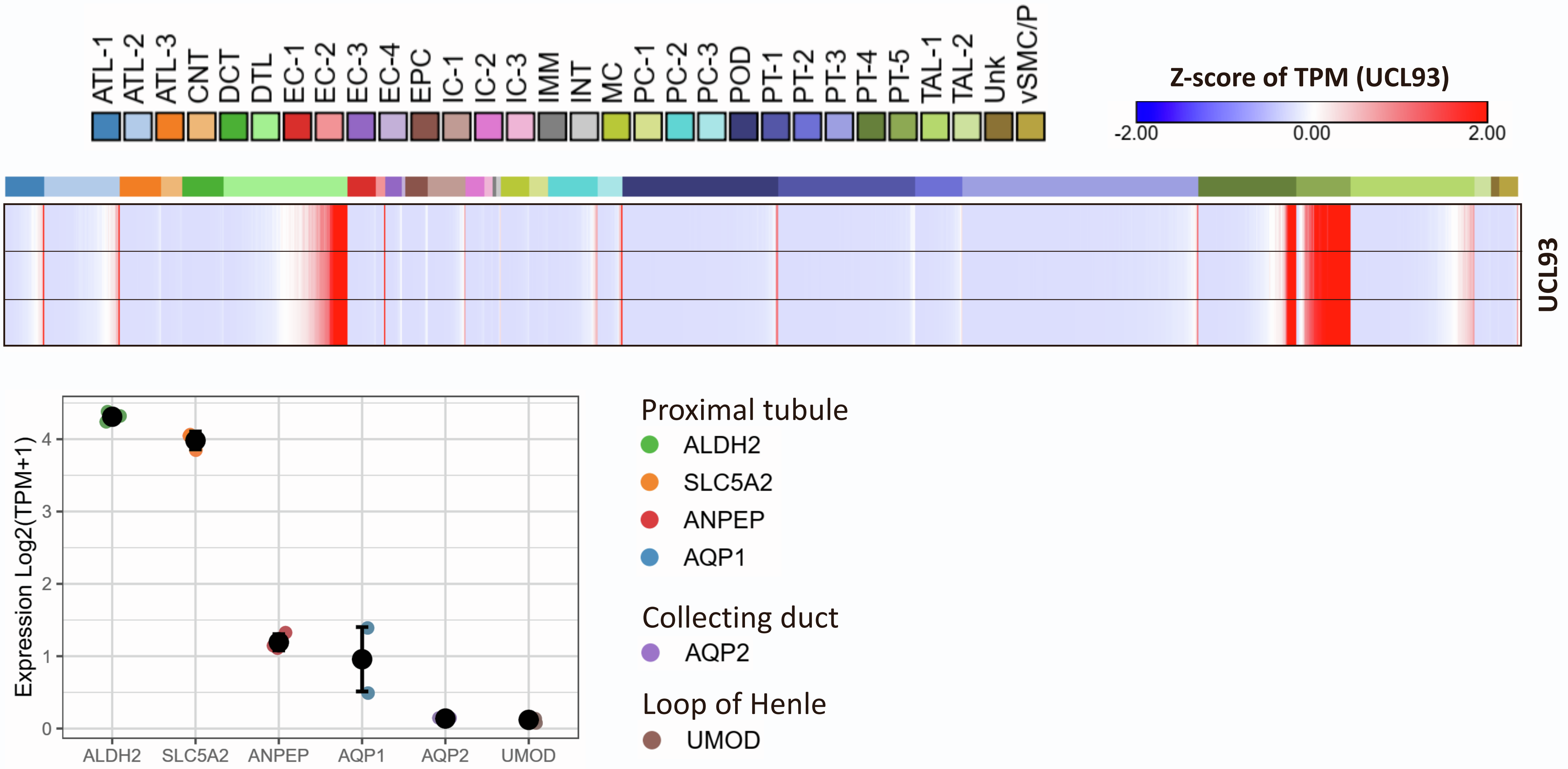
ATL-1
ATL-2
ATL-3
CNT
DCT
DTL
EC-1
EC-2
EC-3
EC-4
EPC
IC-1
IC-2
IC-3
IMM
INT
MC
PC-1
PC-2
PC-3
POD
PT-1
PT-2
PT-3
PT-4
PT-5
TAL-1
TAL-2
Unk
vSMC/P
Z-score of TPM (UCL93)
-2.00
0.00
2.00
UCL93
Expression Log2(TPM+1)
0
1
2
3
4
ALDH2
SLC5A2
ANPEP
AQP1
AQP2
UMOD
Proximal tubule
ALDH2
SLC5A2
ANPEP
AQP1
Collecting duct
AQP2
Loop of Henle
UMOD

Figure S3

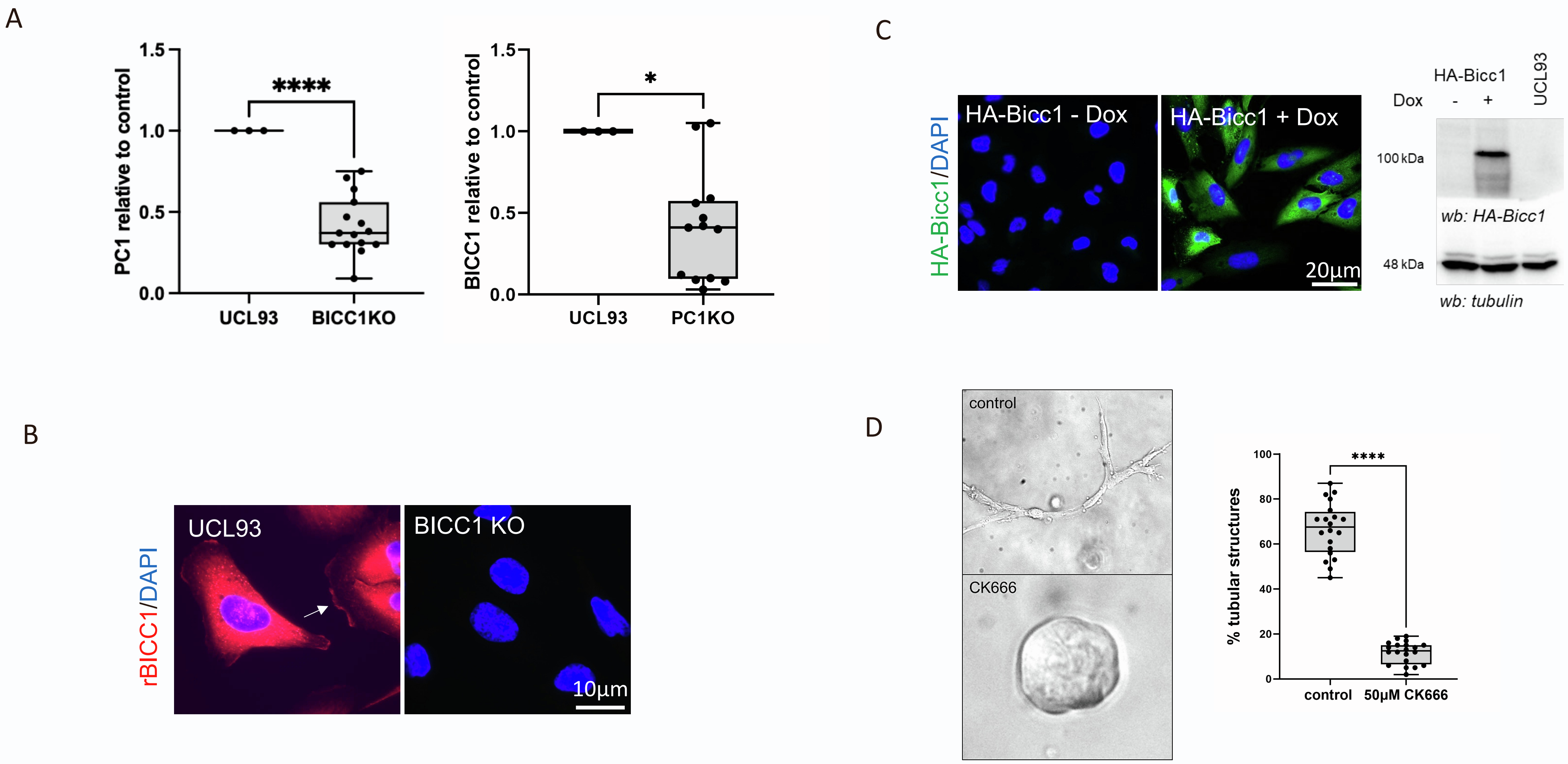

A
PC1 relative to control
1.5
1.0
0.5
0.0
****
UCL93
BICC1KO
BICC1 relative to control
*
PC1KO
C
HA-Bicc1/DAPI
HA-Bicc1 - Dox
HA-Bicc1 + Dox
20µm
HA-Bicc1
Dox
-
+
UCL93
100 kDa
wb: HA-Bicc1
48 kDa
wb: tubulin
B
rBICC1/DAPI
UCL93
BICC1 KO
10µm
D
control
CK666
% tubular structures
100
80
60
40
20
0
****
50µM CK666

Figure S4

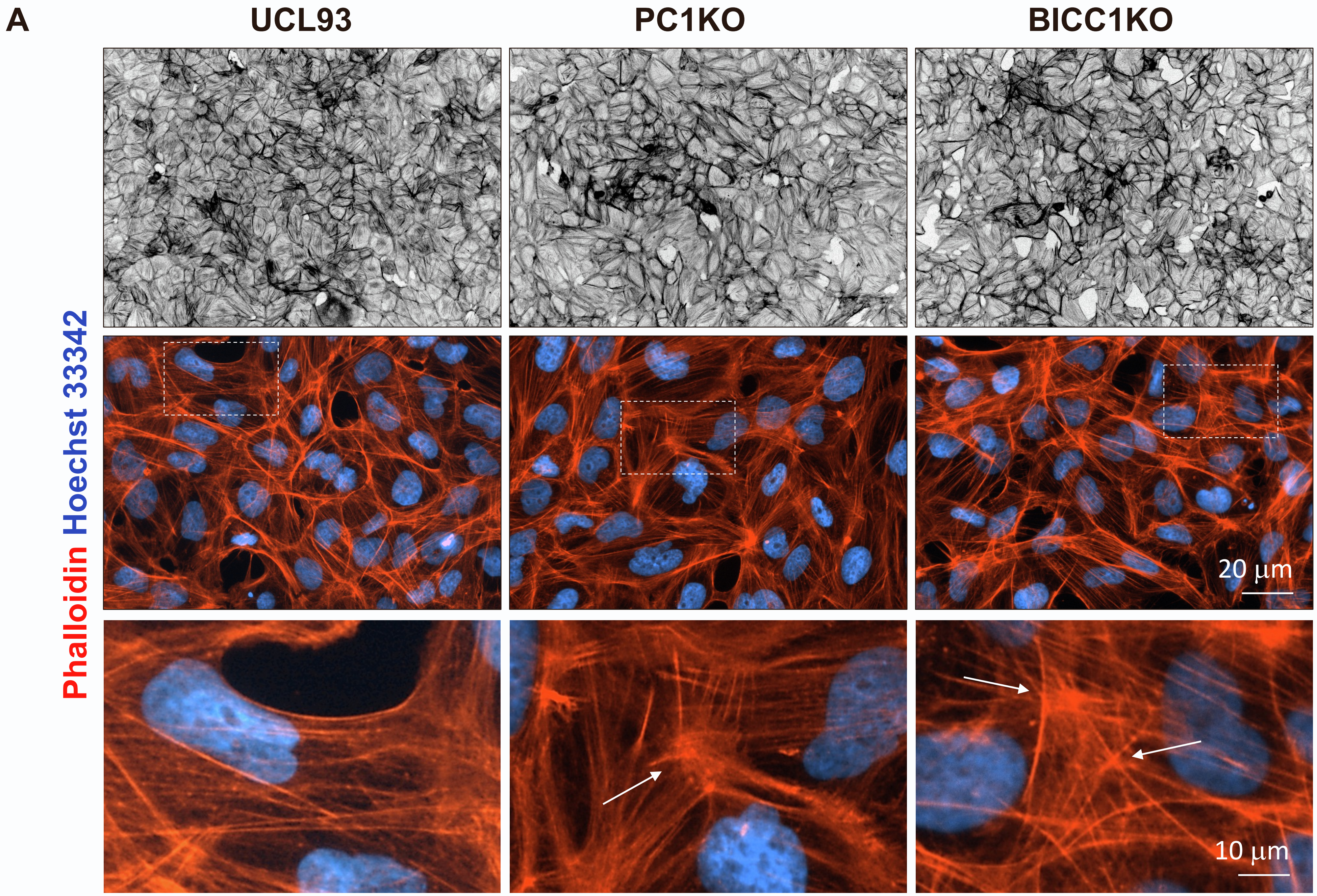

Figure S5

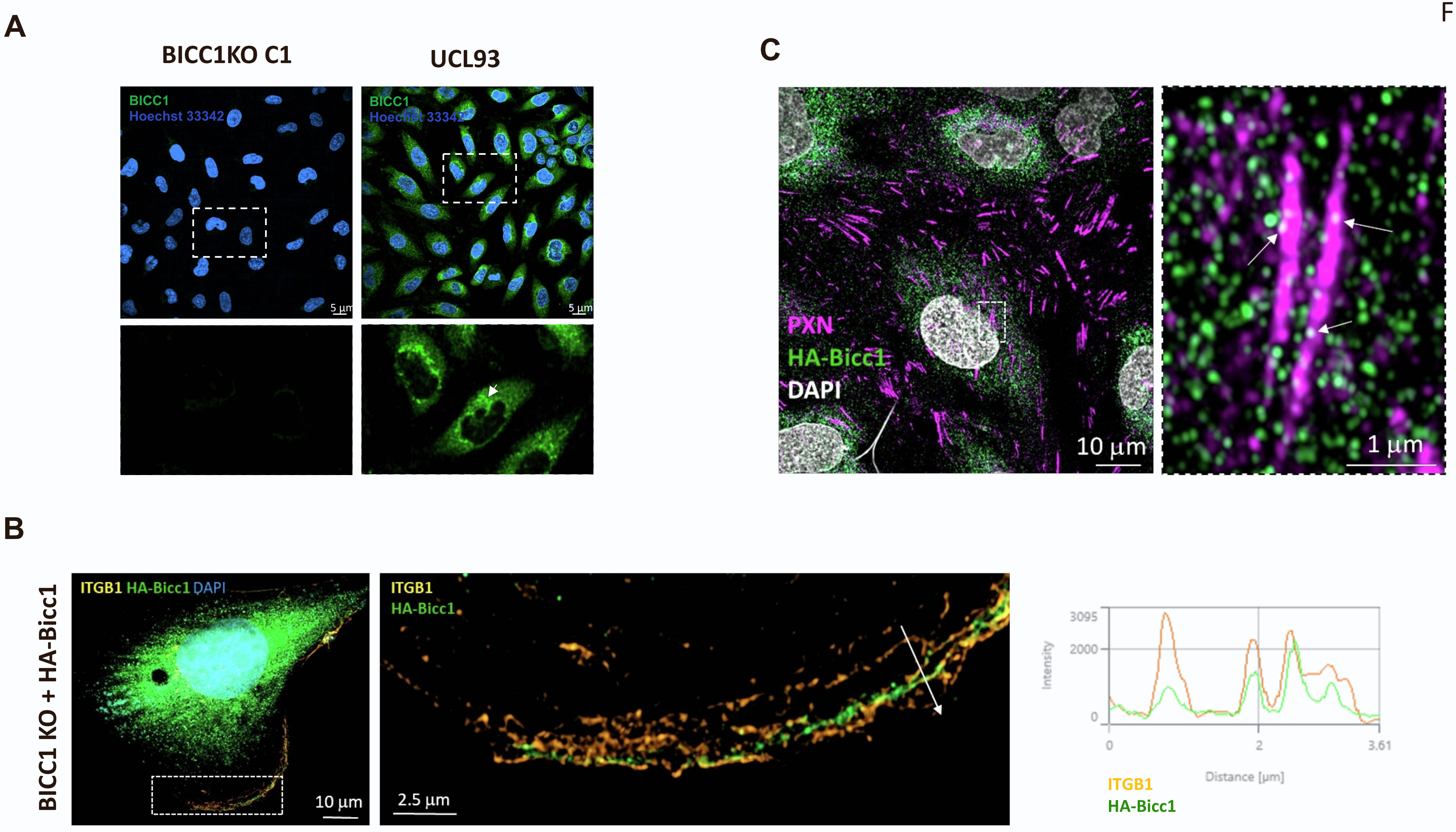

A
BICC1KO C1
UCL93
BICC1
Hoechst 33342
5 µm
C
PXN
HA-Bicc1
DAPI
10 µm
1 µm
B
BICC1 KO + HA-Bicc1
ITGB1 HA-Bicc1 DAPI
10 µm
ITGB1
HA-Bicc1
2.5 µm
3095
2000
0
Intensity
0
2
3.61
Distance [µm]
ITGB1
HA-Bicc1

Figure S6

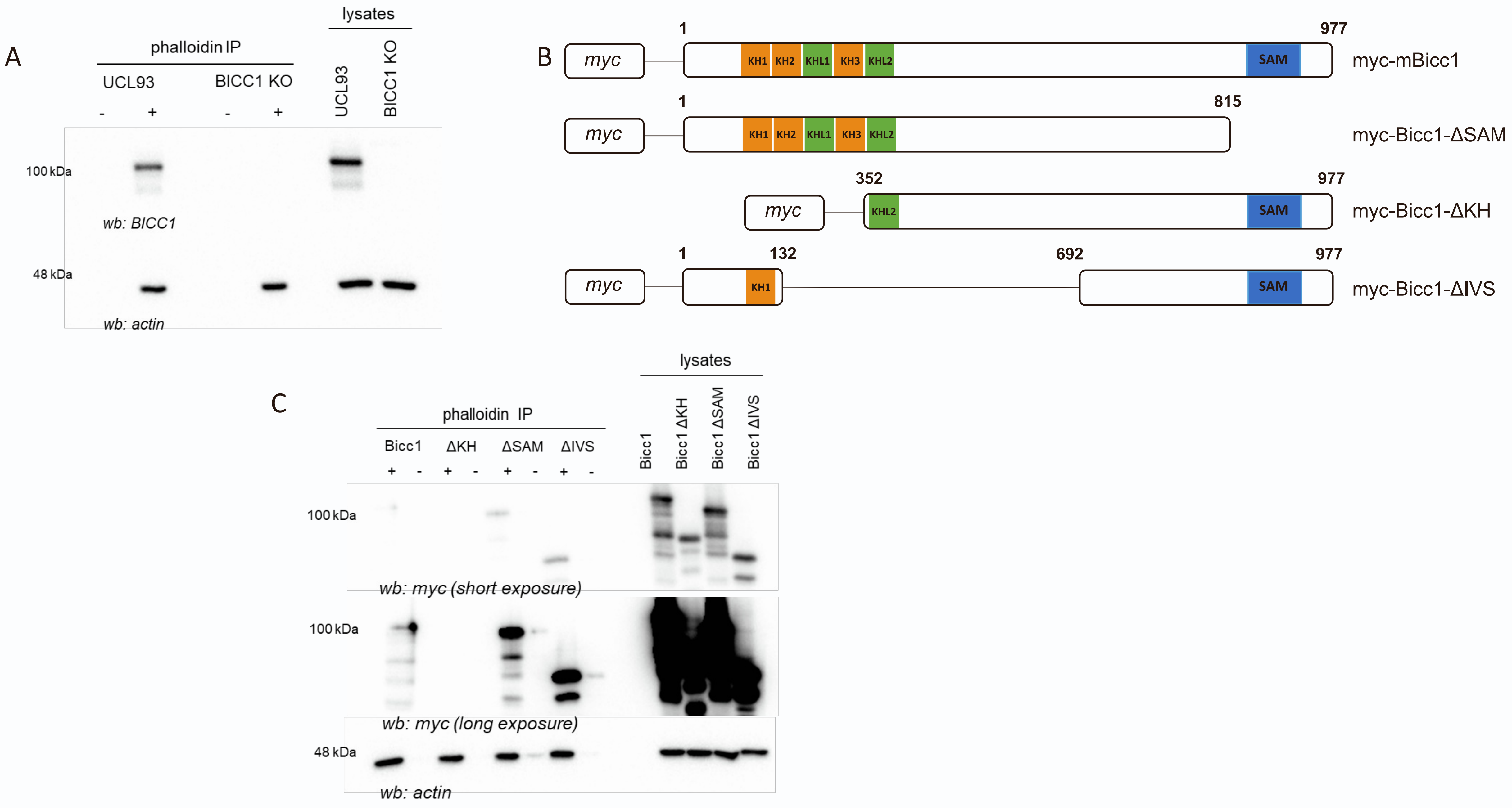
A
phalloidin IP
lysates
UCL93
BICC1 KO
UCL93
BICC1 KO
-
+
-
+
100 kDa
wb: BICC1
48 kDa
wb: actin
B
myc
KH1
KH2
KHL1
KH3
KHL2
SAM
1
977
815
352
132
692
myc-mBicc1
myc-Bicc1-ΔSAM
myc-Bicc1-ΔKH
myc-Bicc1-ΔIVS
C
phalloidin IP
lysates
Bicc1
ΔKH
ΔSAM
ΔIVS
Bicc1 ΔKH
Bicc1 ΔSAM
Bicc1 ΔIVS
100 kDa
wb: myc (short exposure)
wb: myc (long exposure)
48 kDa
wb: actin

Figure S7

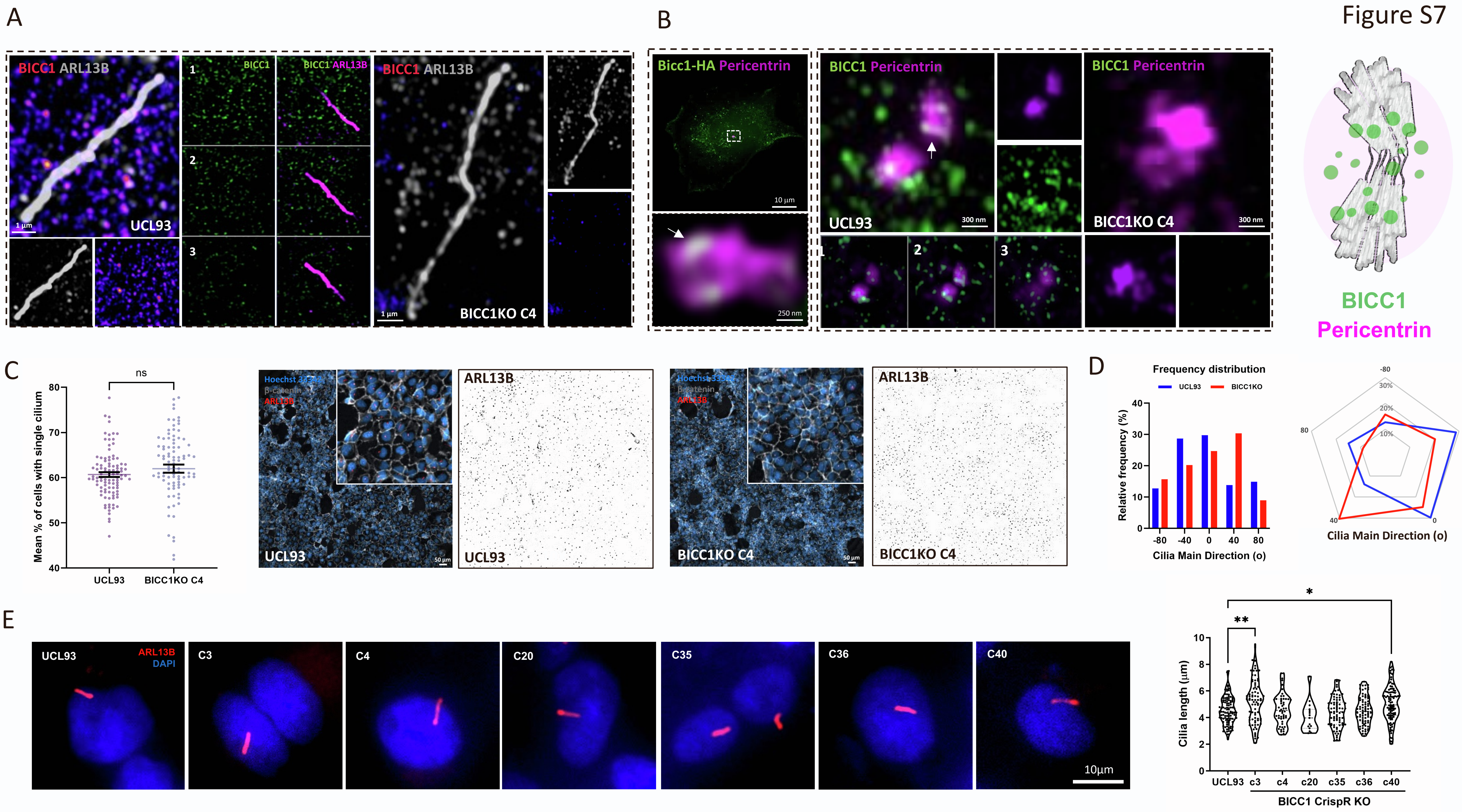
A
BICC1 ARL13B
UCL93
1 μm
1
2
3
BICC1
BICC1 ARL13B
BICC1 ARL13B
BICC1KO C4
1 μm
B
Bicc1-HA Pericentrin
10 μm
250 nm
BICC1 Pericentrin
UCL93
300 nm
BICC1 Pericentrin
BICC1KO C4
300 nm
1
2
3
BICC1
Pericentrin
C
ns
Mean % of cells with single cilium
80
70
60
50
40
UCL93
BICC1KO C4
Hoechst 33342
β-catenin
ARL13B
UCL93
50 μm
ARL13B
UCL93
BICC1KO C4
ARL13B
BICC1KO C4
D
Frequency distribution
UCL93
BICC1KO
Relative frequency (%)
40
30
20
10
0
-80
-40
0
40
80
Cilia Main Direction (o)
-80
30%
20%
10%
-40
0
40
80
Cilia Main Direction (o)
E
UCL93
ARL13B
DAPI
C3
C4
C20
C35
C36
C40
10μm
*
**
Cilia length (μm)
10
8
6
4
2
0
UCL93
c3
c4
c20
c35
c36
c40
BICC1 CrispR KO

Figure S8

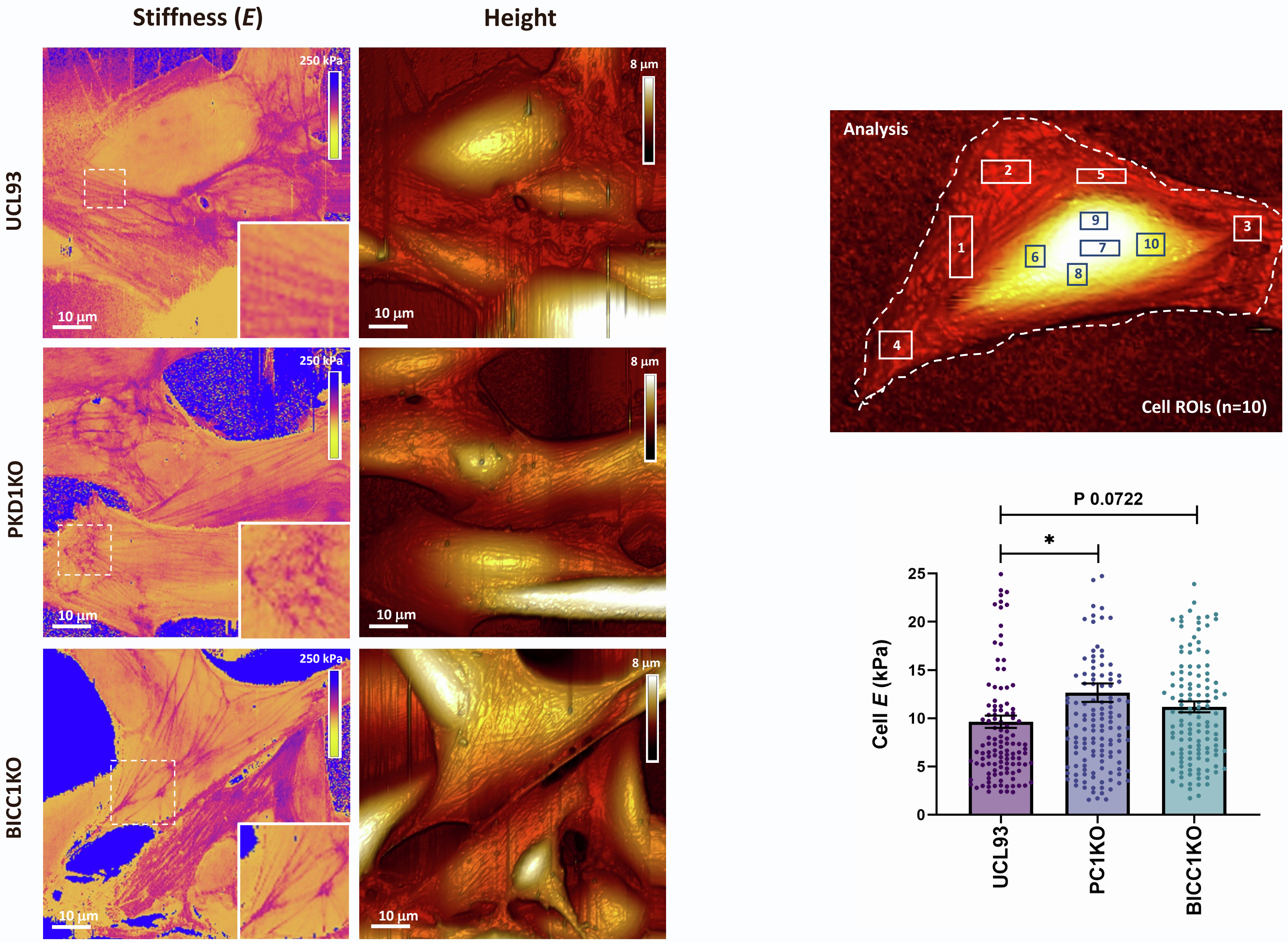

Stiffness (E)
Height
UCL93
PKD1KO
BICC1KO
250 kPa
8 µm
10 µm
Analysis
1
2
3
4
5
6
7
8
9
10
Cell ROIs (n=10)
P 0.0722
*
Cell E (kPa)
0
5
10
15
20
25
UCL93
PC1KO
BICC1KO

Figure S9

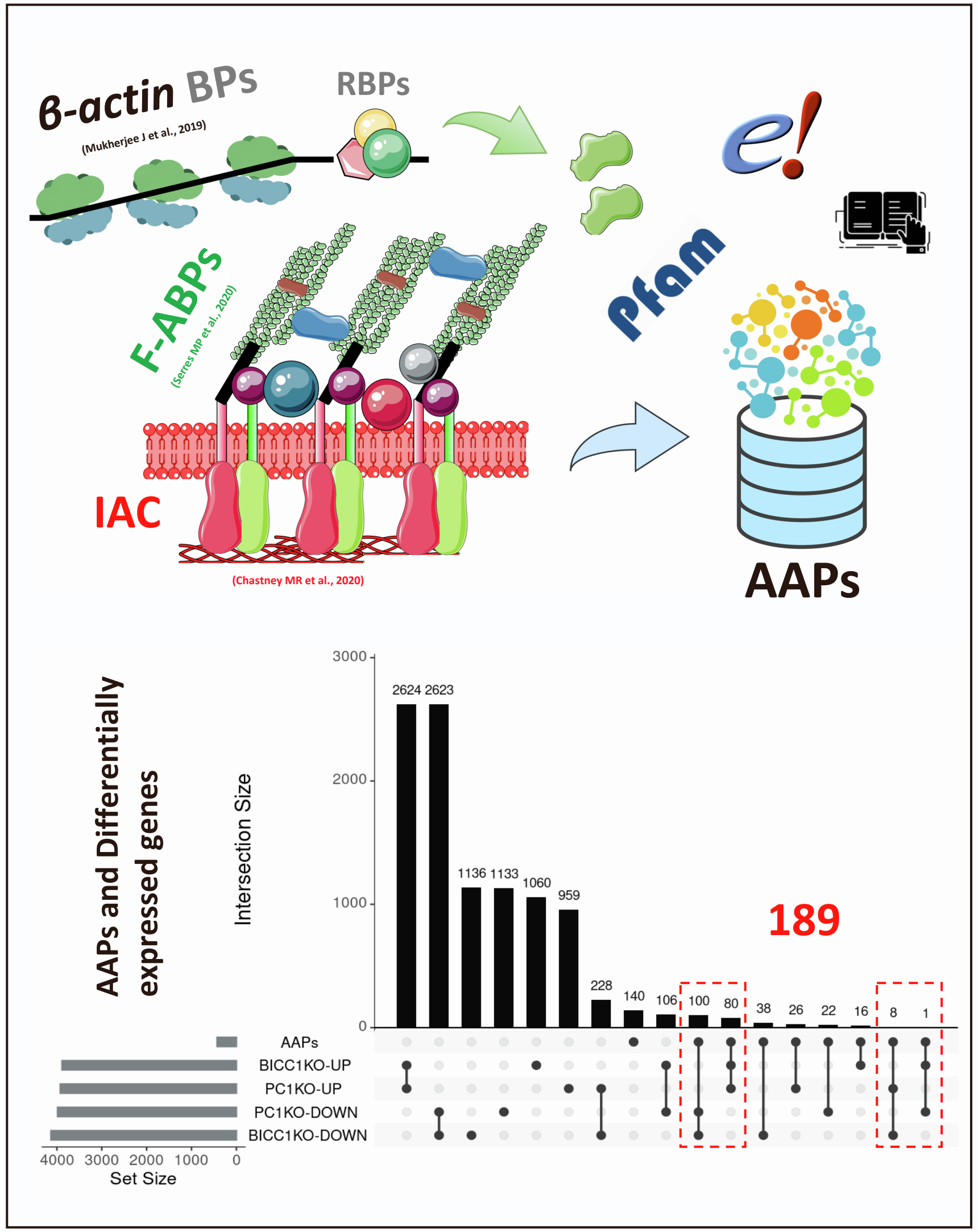

Key References for Actin-Associated Proteins (AAPs) Dataset

Serres MP, Samwer M, Truong Quang BA*, et al.* F-Actin Interactome Reveals Vimentin as a Key Regulator of Actin Organization and Cell Mechanics in Mitosis. *Dev Cell* 2020; **52:** 210-222 e217.

Mukherjee J, Hermesh O, Eliscovich C*, et al.* beta-Actin mRNA interactome mapping by proximity biotinylation. *Proc Natl Acad Sci U S A* 2019; **116:** 12863-12872.

Chastney MR, Lawless C, Humphries JD*, et al.* Topological features of integrin adhesion complexes revealed by multiplexed proximity biotinylation. *J Cell Biol* 2020; **219**.

Figure S10

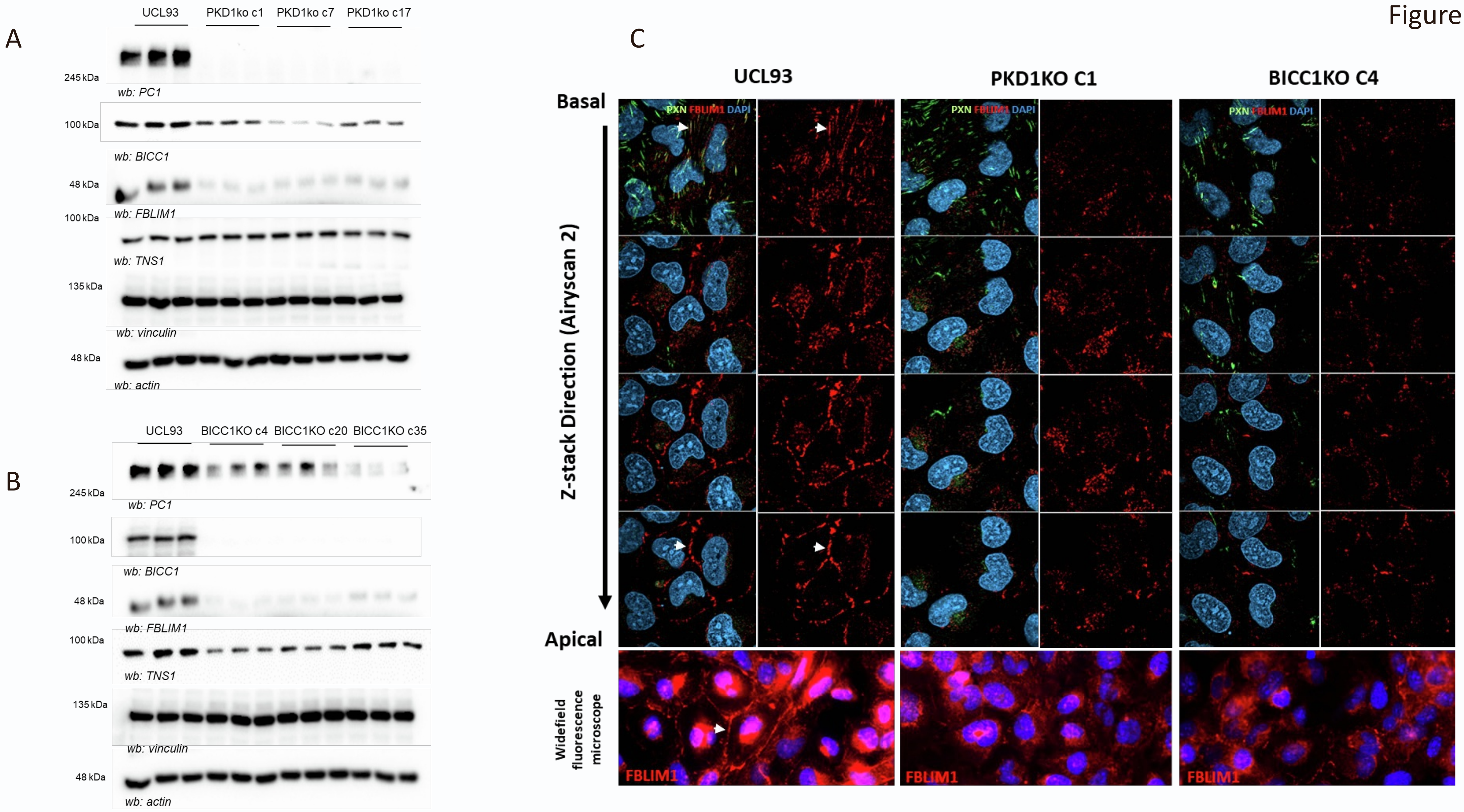
A
UCL93
PKD1ko c1
PKD1ko c7
PKD1ko c17
245 kDa
wb: PC1
100 kDa
wb: BICC1
48 kDa
wb: FBLIM1
100 kDa
wb: TNS1
135 kDa
wb: vinculin
48 kDa
wb: actin
B
UCL93
BICC1KO c4
BICC1KO c20
BICC1KO c35
245 kDa
wb: PC1
100 kDa
wb: BICC1
48 kDa
wb: FBLIM1
100 kDa
wb: TNS1
135 kDa
wb: vinculin
48 kDa
wb: actin
C
UCL93
PKD1KO C1
BICC1KO C4
Basal
Z-stack Direction (Airyscan 2)
Apical
PXN FBLIM1 DAPI
Widefield fluorescence microscope
FBLIM1

Figure S11

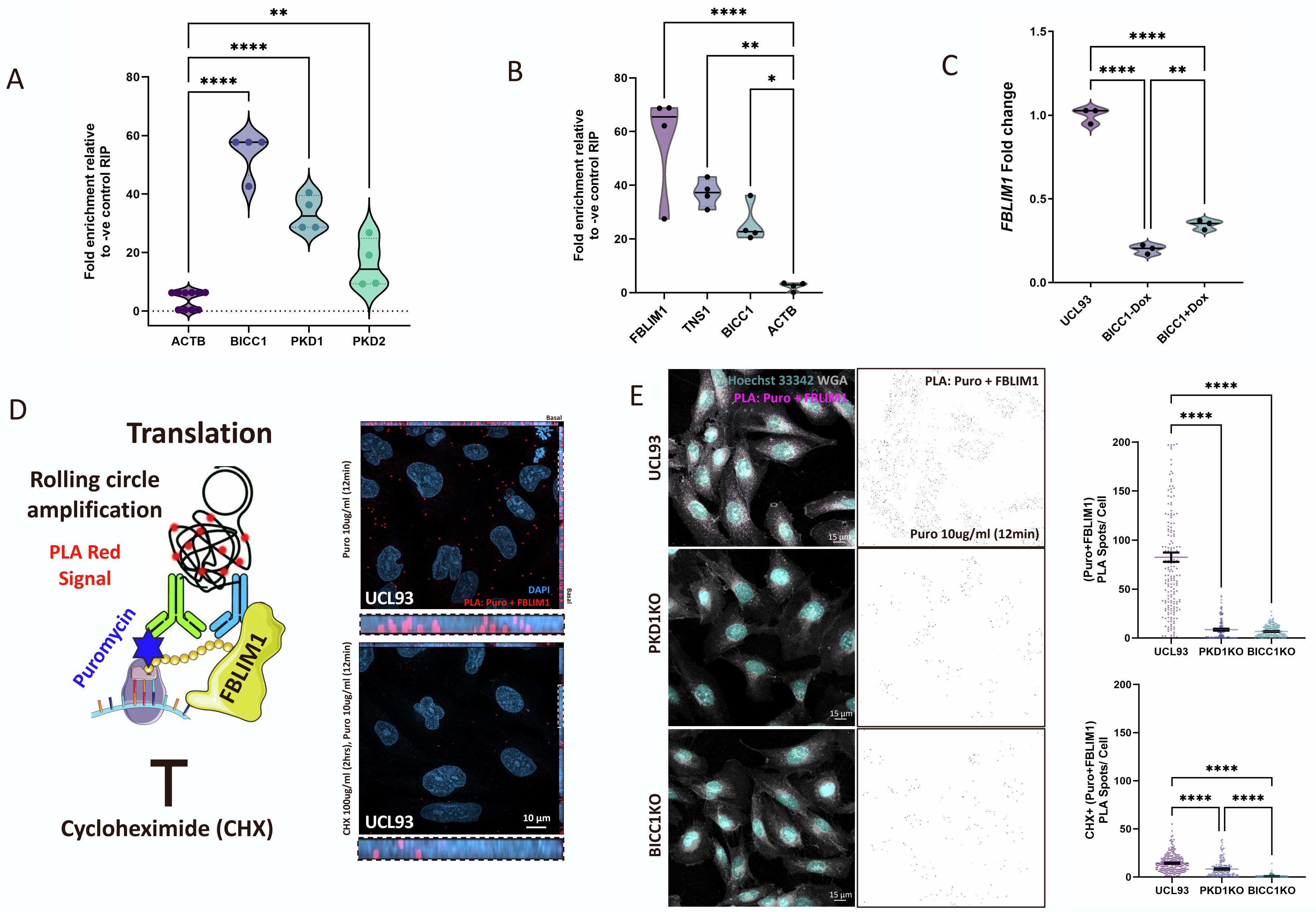

A
Fold enrichment relative to -ve control RIP
ACTB
BICC1
PKD1
PKD2
B
Fold enrichment relative to -ve control RIP
FBLIM1
TNS1
BICC1
ACTB
C
FBLIM1 Fold change
UCL93
BICC1-Dox
BICC1+Dox
D
Translation
Rolling circle amplification
PLA Red Signal
Puromycin
FBLIM1
Cycloheximide (CHX)
Puro 10ug/ml (12min)
UCL93
DAPI
PLA: Puro + FBLIM1
CHX 100ug/ml (2hrs), Puro 10ug/ml (12min)
UCL93
10 µm
E
Hoechst 33342 WGA
PLA: Puro + FBLIM1
PLA: Puro + FBLIM1
Puro 10ug/ml (12min)
UCL93
PKD1KO
BICC1KO
15 µm
(Puro+FBLIM1) PLA Spots/ Cell
UCL93
PKD1KO
BICC1KO
CHX+ (Puro+FBLIM1) PLA Spots/ Cell
UCL93
PKD1KO
BICC1KO

Figure S12

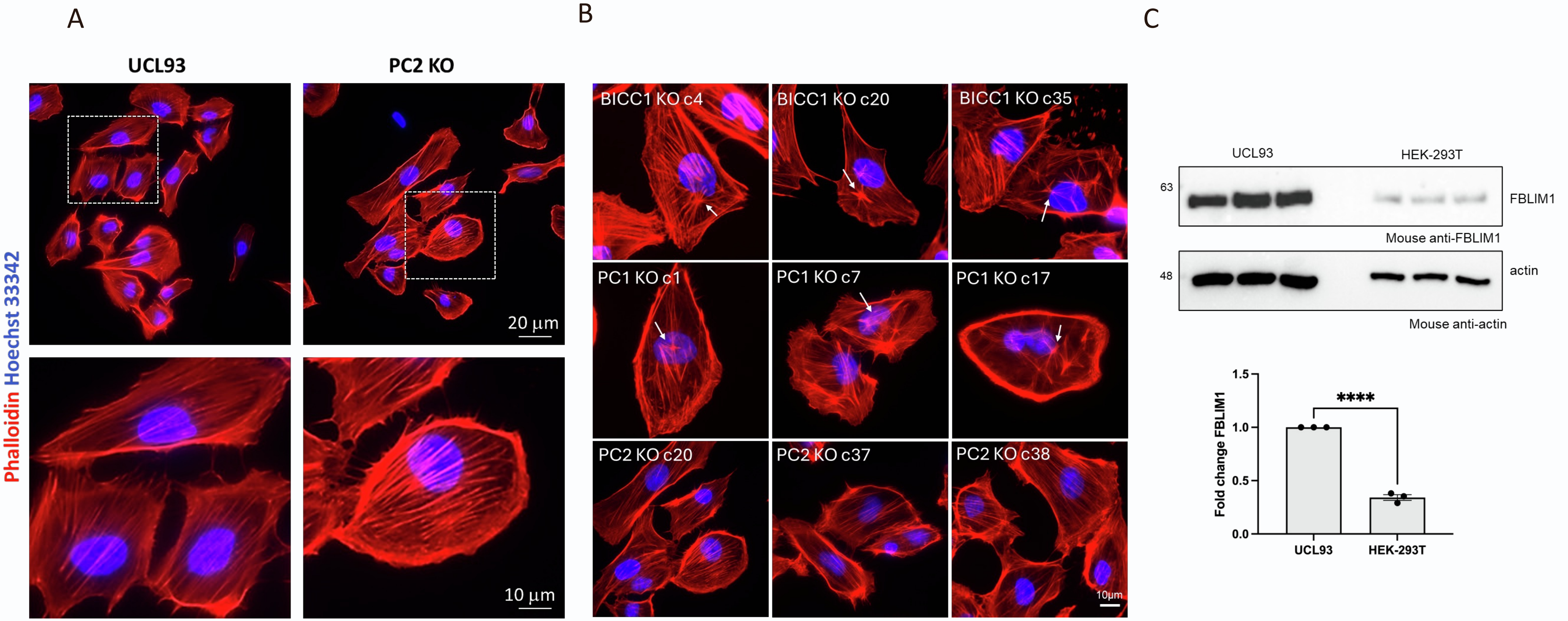
A
UCL93
PC2 KO
Phalloidin Hoechst 33342
20 μm
10 μm
B
BICC1 KO c4
BICC1 KO c20
BICC1 KO c35
PC1 KO c1
PC1 KO c7
PC1 KO c17
PC2 KO c20
PC2 KO c37
PC2 KO c38
10μm
C
UCL93
HEK-293T
63
48
FBLIM1
Mouse anti-FBLIM1
actin
Mouse anti-actin
Fold change FBLIM1
1.5
1.0
0.5
0.0
****
UCL93
HEK-293T
